# A Novel Serum Metabolomics-Based Diagnostic Approach for Colorectal Cancer

**DOI:** 10.1371/journal.pone.0040459

**Published:** 2012-07-11

**Authors:** Shin Nishiumi, Takashi Kobayashi, Atsuki Ikeda, Tomoo Yoshie, Megumi Kibi, Yoshihiro Izumi, Tatsuya Okuno, Nobuhide Hayashi, Seiji Kawano, Tadaomi Takenawa, Takeshi Azuma, Masaru Yoshida

**Affiliations:** 1 Division of Gastroenterology, Department of Internal Medicine, Kobe University Graduate School of Medicine, Kobe, Japan; 2 Department of Clinical Laboratory, Kobe University Hospital, Kobe, Japan; 3 The Integrated Center for Mass Spectrometry, Kobe University Graduate School of Medicine, Kobe, Japan; 4 Division of Metabolomics Research, Department of Internal Medicine, Kobe University Graduate School of Medicine, Kobe, Japan; Baylor College of Medicine, United States of America

## Abstract

**Background:**

To improve the quality of life of colorectal cancer patients, it is important to establish new screening methods for early diagnosis of colorectal cancer.

**Methodology/Principal Findings:**

We performed serum metabolome analysis using gas-chromatography/mass-spectrometry (GC/MS). First, the accuracy of our GC/MS-based serum metabolomic analytical method was evaluated by calculating the RSD% values of serum levels of various metabolites. Second, the intra-day (morning, daytime, and night) and inter-day (among 3 days) variances of serum metabolite levels were examined. Then, serum metabolite levels were compared between colorectal cancer patients (N = 60; N = 12 for each stage from 0 to 4) and age- and sex-matched healthy volunteers (N = 60) as a training set. The metabolites whose levels displayed significant changes were subjected to multiple logistic regression analysis using the stepwise variable selection method, and a colorectal cancer prediction model was established. The prediction model was composed of 2-hydroxybutyrate, aspartic acid, kynurenine, and cystamine, and its AUC, sensitivity, specificity, and accuracy were 0.9097, 85.0%, 85.0%, and 85.0%, respectively, according to the training set data. In contrast, the sensitivity, specificity, and accuracy of CEA were 35.0%, 96.7%, and 65.8%, respectively, and those of CA19-9 were 16.7%, 100%, and 58.3%, respectively. The validity of the prediction model was confirmed using colorectal cancer patients (N = 59) and healthy volunteers (N = 63) as a validation set. At the validation set, the sensitivity, specificity, and accuracy of the prediction model were 83.1%, 81.0%, and 82.0%, respectively, and these values were almost the same as those obtained with the training set. In addition, the model displayed high sensitivity for detecting stage 0–2 colorectal cancer (82.8%).

**Conclusions/Significance:**

Our prediction model established via GC/MS-based serum metabolomic analysis is valuable for early detection of colorectal cancer and has the potential to become a novel screening test for colorectal cancer.

## Introduction

Colorectal cancer is one of the most common causes of cancer death in developed countries [1]. Treatment methods based on colonoscopy and surgery have advanced rapidly, and a large number of patients with colorectal cancer achieve improvements after therapy. However, advanced stage colorectal cancer reduces the quality of life of patients receiving operative treatment or chemotherapy. Therefore, methods that allow the early detection and diagnosis of colorectal cancer are currently being sought. The fecal occult blood test (FOBT) is the most commonly used screening method for diagnosing colorectal cancer and is a noninvasive and inexpensive method. However, the FOBT has low sensitivity, especially for early stage colorectal cancer. Colonoscopy is a more accurate and reliable approach for diagnosing colorectal cancer, but it is difficult for elderly or severely ill patients to undergo colonoscopy, and its high cost is also a problem. Thus, examinations involving a combination of conventional screening methods have been used for the diagnosis of colorectal cancer; however, such examinations only detect about 40% of colorectal cancers [2]. Therefore, it is necessary to establish new screening methods for the early diagnosis of colorectal cancer that are highly sensitive, specific, easy, and noninvasive.

The human genome had been completely identified by the end of 2003. Since then, proteomics, which is the comprehensive study of the entire set of proteins expressed by a genome, has been extensively studied, and many researchers have tried to apply proteomic analysis to the medical field in order to find effective diagnostic markers and elucidate unknown pathological conditions [3]. Recently, metabolomics, which is the comprehensive study of low molecular weight metabolites, has also been developed. In clinical research involving metabolome analysis using a combination of high-throughput liquid-chromatography/mass-spectrometry (LC/MS) and gas-chromatography/mass-spectrometry (GC/MS), Sreekumar et al. demonstrated that sarcosine is a potentially important metabolic intermediary for prostate cancer cell invasion and aggressivity [4]. In addition, a comprehensive and quantitative analysis of the charged metabolites in tumor and normal tissues obtained from colorectal and gastric cancer patients was performed using capillary electrophoresis-mass spectrometry (CE/MS) [5]. Thus, various types of clinical samples have been analyzed by metabolome analysis using nuclear magnetic resonance (NMR), GC/MS, LC/MS, CE/MS, and/or matrix assisted laser desorption ionization-mass spectrometry (MALDI-MS) in order to elucidate disease onset mechanisms and discover novel biomarkers [6]. Among these techniques, GC/MS has a long history and is easier to use than CE/MS or MALDI-MS, although GC/MS has low sensitivity compared with LC/MS. Moreover, there are more databases of GC/MS-based serum metabolite analysis results than of LC/MS-based serum metabolite analysis results. In addition, GC/MS can be applied to large-scale studies with relative ease due to its high repeatability. Therefore, in this study, the serum metabolite levels of colorectal cancer patients and healthy volunteers were analyzed by GC/MS analysis to establish new diagnostic tools for colorectal cancer, and the stability and inter-day and intra-day variances of these serum metabolite levels were also evaluated. Using a training set composed of colorectal cancer patients (N = 60) and healthy volunteers (N = 60), a colorectal cancer-prediction model was established via multiple logistic regression analysis using the stepwise variable selection method. Then, the validity of the prediction model was assessed using a validation set consisting of colorectal cancer patients (N = 59) and healthy volunteers (N = 63).

## Methods

### Ethics and Participants

This study was approved by the ethics committee at Kobe University Graduate School of Medicine, and performed between Feb. 2009 and Dec. 2011. The human samples were used in accordance with the guidelines of Kobe University Hospital, and written informed consent was obtained from all subjects. To calculate the relative standard deviation (RSD)% for the serum metabolome analysis results obtained by GC/MS, blood samples were collected from one healthy 30-year-old male volunteer after fasting in the early morning, and the serum was separated by centrifugation at 3,000 x g for 10 min at 4°C. The serum was transferred to a clean tube and stored at −80°C until use. To evaluate the intra-day variance in serum metabolite levels, whole blood samples were collected from healthy volunteers (n = 16) at 8:00 a.m.–9:00 a.m. before breakfast, 12:00 p.m.–1:00 p.m. before lunch, and 6:00 p.m.–7:00 p.m. before dinner. To evaluate the inter-day variance in serum metabolite levels, whole blood samples (n = 16) were collected at 8:00 a.m.–9:00 a.m. before breakfast once a day for a total of 3 days. For the training set, 60 serum samples each were obtained from colorectal cancer patients and healthy volunteers after fasting in the early morning. For the validation set, 59 and 63 serum samples were collected from colorectal cancer patients and healthy volunteers, respectively. The serum samples from the colorectal cancer patients were collected at Kobe University Hospital. None of the cancer patients had any complicating diseases. The patients were diagnosed by microscopy, biopsy, or surgical resection and classified using the sixth edition of the International Union Against Cancer classification (UICC). The serum samples from the healthy volunteers were obtained from Kobe University Hospital and two other facilities. In Kobe University Hospital, it was confirmed that there is no abnormality of blood tests, endoscopic examinations, diagnostic imaging, and/or medical interview. At two other facilities, healthy volunteers were selected via health checks including blood tests, endoscopic examinations, diagnostic imaging, and/or medical interviews. Individuals that had been diagnosed as requiring therapy, detailed examinations, and/or observations were not treated as healthy volunteers. The characteristics of all subjects are summarized in Table 1, Table S1, and Table S2.

**Table 1:** Subject information for the training and validation sets.

		Training set			Validation set		
		Colorectal cancer patients	Healthyvolunteers	Significance	Colorectal cancer patients	Healthyvolunteers	Significance
**N**		60	60		59	63	
	**Male**	39	39		30	32	
	**Female**	21	21		29	31	
**Age**	**(years)**						
	**Mean**	67.7	64.5	N.S.	64.8	62.8	N.S.
	**Median**	70	68		66	63	
	**Range**	36–88	39–88		31–84	47–73	
**BMI**	**(%)**	21.9	22.1	N.S.	22.5	22.2	N.S.
							
**TNM stage**	**Zero**	12	−		15	−	
	**I**	12	−		11	−	
	**II**	12	−		3	−	
	**III**	12	−		11	−	
	**IV**	12	−		19	−	
**Cancer location**	**Ascending colon**	9	−		8	−	
	**Transverse colon**	9	−		4	−	
	**Descending colon**	2	−		3	−	
	**Sigmoid colon**	14	−		18	−	
	**Cecum**	5	−		9	−	
	**Rectum**	21	−		17	−	

The differences in the mean age and BMI value (%) between the colorectal cancer patients and healthy volunteers were evaluated using the Mann-Whitney U test. N.S., not significant.

### Experimental Procedures

The extraction of low molecular weight metabolites was performed according to the method described in our previous report [7]. Briefly, 50 µl of serum were mixed with 250 µl of a solvent mixture (MeOH:H_2_O:CHCl_3_ = 2.5:1:1) containing 10 µl of 0.5 mg/ml 2-isopropylmalic acid (Sigma-Aldrich, Tokyo, Japan) dissolved in distilled water as an internal standard, and then the solution was shaken at 1,200 rpm for 30 min at 37°C, before being centrifuged at 16,000 x g for 3 min at 4°C. Two hundred and twenty-five µl of the resultant supernatant were transferred to a clean tube, and 200 µl of distilled water were added to the tube. After being mixed, the solution was centrifuged at 16,000 x g for 3 min at 4°C, and 250 µl of the resultant supernatant were transferred to a clean tube, before being lyophilized using a freeze dryer. For oximation, 40 µl of 20 mg/ml methoxyamine hydrochloride (Sigma-Aldrich, Tokyo, Japan) dissolved in pyridine were mixed with a lyophilized sample, before being shaken at 1,200 rpm for 90 min at 30°C. Next, 20 µl of N-methyl-N-trimethylsilyl-trifluoroacetamide (MSTFA) (GL Science, Tokyo, Japan) were added for derivatization, and the mixture was incubated at 1,200 rpm for 30 min at 37°C. The mixture was then centrifuged at 16,000 x g for 5 min at 4°C, and the resultant supernatant was subjected to GC/MS measurement.

According to the method describe in a previous report [8], GC/MS analysis was performed using a GCMS-QP2010 Ultra (Shimadzu Co., Kyoto, Japan) with a fused silica capillary column (CP-SIL 8 CB low bleed/MS; 30 m × 0.25 mm inner diameter, film thickness: 0.25 µm; Agilent Co., Palo Alto, CA). The front inlet temperature was 230°C. The flow rate of helium gas through the column was 39.0 cm/sec. The column temperature was held at 80°C for 2 min and then raised by 15°C/min to 330°C and held there for 6 min. The transfer line and ion-source temperatures were 250°C and 200°C, respectively. Twenty scans per second were recorded over the mass range 85–500 m/z using the Advanced Scanning Speed Protocol (ASSP, Shimadzu Co.). In this study, the detection voltage was confirmed every day before GC/MS analysis, because this value reflects on the degree of contamination in the instrument. In addition, the blank samples were measured before measurement of the serum samples. During GC/MS analysis, the 20 samples per 1 day were measured, and the septum and glass liner in the GC inlet were changed every 100 injections to the column.

Data processing was performed according to the methods described in previous reports [8], [9]. Briefly, MS data were exported in netCDF format. The peak detection and alignment were performed using the MetAlign software (Wageningen UR, The Netherlands). The resultant data were exported in CSV format and then analyzed with in-house analytical software (AIoutput). This software enables peak identification and semi-quantification using an in-house metabolite library. For semi-quantification, the peak height of each ion was calculated and normalized to the peak height of 2-isopropylmalic acid as an internal standard. Names were assigned to each metabolite peak based on the method described in a previous report [9]. All data obtained from the serum samples were subjected to MetAlign software at once, because the same alignment conditions needed to be performed during all data analysis. In GC/MS analysis, multiple peaks are sometimes detected for a particular metabolite due to TMS-derivatization, isomeric form, etc. In such cases, the peak that most reflected the level of the metabolite was adopted for the semi-quantitative evaluation.

### Statistical Analysis

The patients (N = 119) were allocated to the training and validation sets as follows. The colorectal cancer patient samples for the training set were collected without preset selection criteria, and 12 colorectal cancer patients were selected for each disease stage (N = 60). As for the healthy volunteers used for the training set, age- and sex-controlled samples were prepared (N = 60). In the training set study, the levels of metabolites were compared between colorectal cancer patients and healthy volunteers using the Mann-Whitney U test. Among the metabolites that displayed significantly different levels among the groups (*p*<0.05), we selected the metabolites with RSD% values of no more than 20% and that did not display significant intra-day or inter-day variances according to the Wilcoxon signed-rank test and Steel-Dwass test, respectively. The selected metabolites were subjected to a stepwise variable selection method followed by multiple logistic regression analysis, and these analyses were performed using the default conditions of JMP9 (SAS Institute Inc., Cary, NC). The multicollinearity of the metabolites selected via the stepwise variable selection method was confirmed by calculating their variance inflation factors (VIF). AICc, which is Akaike’s Information Criterion (AIC) with a correction for finite sample sizes, was calculated to elucidate the optimal number of factors to include in the predictive model. Nagelkerke R^2^ was also calculated to evaluate the fitness of the multivariate logistic model. Receiver operating characteristic (ROC) analysis was carried out using JMP9 (SAS Institute Inc.), and the optimal cut-off value and AUC, specificity, sensitivity, and accuracy were calculated. In the validation set study, the prediction model was re-evaluated using different samples, and the specificity, sensitivity, and accuracy of the model were examined using the cut-off value obtained from the training set. *p* values of less than 0.05 were considered to indicate a significant difference.

## Results

In our GC/MS-based metabolomic analysis system, which mainly targeted water-soluble metabolites, 132 metabolites were detected in the subjects’ sera (Table S3). Among the 132 metabolites, 1 metabolite; i.e., 2-isopropylmalic acid, was used as an internal standard, and 5 metabolites were probably extracted from non-serum, for example, they were derived from eppendorf tubes. Therefore, these 6 metabolites were excluded from the subsequent analyses. Since oxalacetic acid was converted to pyruvate during the pre-treatment procedure, oxalacetic acid was detected as pyruvate in our system. Therefore, it is described as ‘pyruvate+oxalacetic acid’ in the Supporting tables and as ‘pyruvate’, which was actually detected by GC/MS analysis, in this manuscript. Due to their similar structures, citric acid and isocitric acid were detected at the same retention time by our system. Therefore, they are described as ‘citric acid+isocitric acid’. Since cysteamine was converted to cystamine during the pre-treatment procedure, cysteamine was detected as cystamine by our system. Therefore, it is described as ‘cysteamine+cystamine’ in the Supporting tables and as ‘cystamine’, which was actually detected by GC/MS analysis, in this manuscript. Since cysteine was converted to cystine during the pre-treatment procedure, cysteine was detected as cystine by our system. Therefore, it is described as ‘cysteine+cystine’ in the Supporting tables and as ‘cystine’, which was actually detected by GC/MS analysis, in this manuscript.

**Figure 1:**
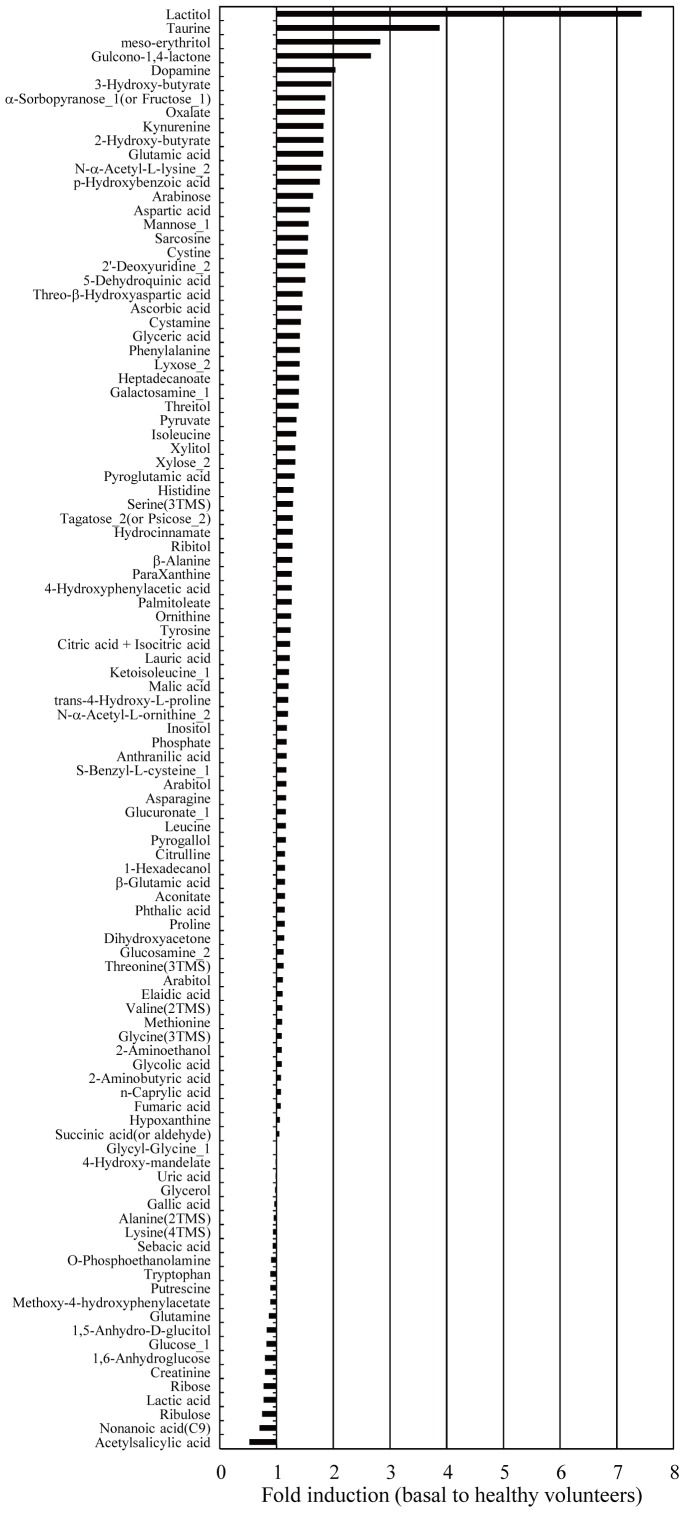
Comparison of serum metabolite levels between colorectal cancer patients and healthy volunteers. The mean fold changes in the levels of 107 metabolites observed in a comparison between colorectal cancer patients (N = 60) and healthy volunteers (N = 60) (training set) are shown in Figure 1. In GC/MS analysis, multiple peaks are sometimes detected for a particular metabolite due to TMS-derivatization, isomeric form, etc. In such cases, the peak that most reflected the level of the metabolite was adopted for the subsequent evaluation. In addition, each metabolite had the term ‘_1’, ‘_2’, or ‘(-TMS)’ added to the end of its name, as described in a previous report [7]. This figure does not include background metabolites or minor peak-derived metabolites.

To evaluate the stability of this system using human serum, the serum levels of various metabolites were separately analyzed using serum samples (N = 10) obtained from 1 healthy volunteer (male, 30 years old), and then the RSD% values of the metabolites were calculated (Table S3). The percentage of metabolites with RSD% values of less than 20% and 30% was 68.5% and 86.5%, respectively. Next, the inter-day (among 3 days) and intra-day (morning, daytime, and night) variances of the serum metabolites were evaluated using the Wilcoxon signed-rank test and Steel-Dwass test, respectively (Table S3), because the significant intra-day and/or inter-day variance is likely to lead to the low sensitivity and specificity at the clinical use. Many metabolites did not display significant inter-day and intra-day variances, but 30 metabolites, for example dihydroxyacetone and tryptophan, demonstrated significant variations. In GC/MS analysis, multiple peaks are sometimes detected for a particular metabolite due to TMS-derivatization, isomeric form, etc. In such cases, the peak that most reflects the level of the metabolite was adopted for the subsequent evaluation. For these metabolites, the terms ‘_1’, ‘_2’, and ‘(-TMS)’ were added to the ends of their names according to the method of a previous report [9]. The excluded peaks are indicated by the term ‘Minor’ in Table S4, and after excluding the ‘Minor’ peaks a total of 107 metabolites had their levels compared between colorectal cancer patients and healthy volunteers (Figure 1, Table S4).

In the training set study, the serum metabolite levels of the colorectal cancer patients and health volunteers were compared using the Mann-Whitney U test (Table S4). The training set was composed of colorectal cancer patients (N = 60) and age- and sex-matched healthy volunteers (N = 60) (Table 1, Table S1). There was no significant difference in the mean age or BMI values of the two groups (Table 1). In this study, colorectal cancer was also classified into 2 groups; i.e., group 1 included stage 0, 1, and 2 disease (absence of invasion and metastasis) and group 2 included stage 3 and 4 disease (presence of invasion and metastasis). From the results of the GC/MS analysis, 27 metabolites that met the following conditions were selected as biomarker candidates: an RSD% value of <20%; no significant (p≥0.05) intra-day or inter-day variances according to the Wilcoxon signed-rank test and Steel-Dwass test; and the presence of a significant difference (p<0.05) between the levels of the colorectal cancer patients and healthy volunteers according to the Mann-Whitney U test. ROC curves were produced using the data for these 27 metabolites, (Figure S1), and the cut-off value, AUC, sensitivity, specificity, and accuracy of each metabolite were calculated (Table 2). As a result, nonanoic acid (C9) and p-hydroxybenzoic acid displayed relatively high sensitivity, with values of 86.7% and 90%, respectively. Regarding specificity, cystamine and cystine both exhibited values of 90%, and ornithine (86.7%), citrulline (85.0%), and palmitoleate (85.0%) also demonstrated relatively high specificity. However, there were no metabolites with accuracy values of greater than 80%, suggesting the necessity of performing evaluations using multiple metabolite biomarkers.

**Table 2:** The sensitivity and specificity of the metabolites whose levels differed significantly between the colorectal cancer patients and healthy volunteers.

	Training set					Validation set		
Compounds	Sensitivity	Specificity	Accuracy	AUC (95% CI)	Cut-off	Sensitivity	Specificity	Accuracy
Pyruvate	75.0	61.7	68.3	0.6888 (0.5866–0.7755)	0.31200	72.9	69.8	71.3
2-hydroxy-butyrate	56.7	83.3	70.0	0.7418 (0.6448–0.8199)	0.00965	55.9	69.8	63.1
Phosphate	60.0	68.3	64.2	0.6303 (0.5256–0.7240)	0.45960	49.2	61.9	55.7
Isoleucine	76.7	53.3	65.0	0.6719 (0.5695–0.7603)	0.02162	69.5	52.4	60.7
Nonanoic acid(C9)	86.7	46.7	66.7	0.6568 (0.5515–0.7489)	0.00483	25.4	61.9	44.3
β-alanine	75.0	46.7	60.8	0.6153 (0.5106–0.7102)	0.00192	74.6	38.1	55.7
meso-erythritol	73.3	56.7	65.0	0.6571 (0.5537–0.7477)	0.01524	66.1	57.1	61.5
Aspartic acid	65.0	80.0	72.5	0.7001 (0.5957–0.7870)	0.03750	69.5	71.4	70.5
Pyroglutamic acid	61.7	76.7	69.2	0.7333 (0.6348–0.8127)	0.53590	59.3	79.4	69.7
Creatinine	71.7	63.3	67.5	0.6517 (0.5460–0.7442)	0.00094	44.1	54.0	49.2
Glutamic acid	76.7	66.7	71.7	0.7547 (0.6556–0.8326)	0.10640	78.0	65.1	71.3
p-hydroxybenzoic acid	90.0	65.0	77.5	0.7660 (0.6630–0.8446)	0.00084	81.4	57.1	68.9
Arabinose	76.7	70.0	73.3	0.7860 (0.6922–0.8573)	0.00206	69.5	68.3	68.9
Ribulose	51.7	73.3	62.5	0.6093 (0.5041–0.7055)	0.00253	49.2	36.5	42.6
Asparagine	66.7	55.0	60.8	0.6054 (0.5001–0.7016)	0.01345	61.0	50.8	55.7
Xylitol	56.7	78.3	67.5	0.6749 (0.5716–0.7642)	0.00252	61.0	69.8	65.6
O-phosphoethanolamine	76.7	51.7	64.2	0.6276 (0.5224–0.7217)	0.00209	33.9	65.1	50.0
Ornithine	38.3	86.7	62.5	0.6450 (0.5413–0.7367)	0.04155	42.4	85.7	64.8
Citrulline	41.7	85.0	63.3	0.6058 (0.4967–0.7054)	0.00459	49.2	76.2	63.1
Glucuronate_1	71.7	58.3	65.0	0.6575 (0.5532–0.7485)	0.00470	47.5	84.1	66.4
Glucosamine_2	70.0	70.0	70.0	0.6931 (0.5868–0.7817)	0.00308	80.0	69.8	74.6
Palmitoleate	40.0	85.0	62.5	0.6071 (0.5022–0.7032)	0.00420	35.6	81.0	59.0
Inositol	55.0	70.0	62.5	0.6260 (0.5209–0.7196)	0.14210	44.1	73.0	59.0
Kynurenine	70.0	80.0	75.0	0.8018 (0.7126–0.8686)	0.00109	67.8	73.0	70.5
Cystamine	55.0	90.0	72.5	0.7497 0.6522–0.8267)	0.03885	49.2	79.4	64.8
Cystine	46.7	90.0	68.3	0.7192 (0.6196–0.8011)	0.04477	55.9	93.7	75.4
Lactitol	48.3	78.3	63.3	0.6139 (0.5088–0.7093)	0.00036	40.7	77.8	59.8

The sensitivity, specificity, accuracy, AUC (95% CI), and cut-off values were calculated from the training set data by ROC analysis, as shown in Figure S1. When the validation set was used, sensitivity, specificity, and accuracy were evaluated using the cut-off value obtained from the training set.

As a new evaluation method, the applicability of a multiple logistic regression model involving multiple metabolite biomarkers was examined. Among the 27 metabolites, the 10 metabolites that displayed the greatest differences between their levels in the colorectal cancer patients and those in the healthy volunteers and had the higher levels in the colorectal cancer patients compared with the healthy volunteers were selected: cystamine, cystine, aspartic acid, arabinose, p-hydroxybenzoic acid, glutamic acid, 2-hydroxybutyrate, kynurenine, meso-erythritol, and lactitol (Table 3). Most of these metabolites displayed significantly-altered levels in the stage 0–4 (N = 60), stage 0–2 (N = 36), and stage 3–4 (N = 24) groups. Among these metabolites, arabinose, meso-erythritol, and lactitol are frequently consumed in the diet. Therefore, the remaining 7 metabolites were subjected to a stepwise variable selection method. As a result, 2-hydroxybutyrate, aspartic acid, kynurenine, and cystamine were selected. These 4 metabolites did not display multicollinearity (data not shown). Then, a multiple logistic regression model for predicting colorectal cancer was established on the basis of the data for these metabolites (Table 4).

**Table 3:** Biomarker candidates subjected to multivariate analysis using the stepwise variable selection method.

	Stage 0–4		Stage 0–2		Stage 3–4		
Compounds	Fold induction	p value	Fold induction	p value	Fold induction	p value	Chemical class
**Training set**							
Cystamine	1.43	<0.0001	1.57	<0.0001	1.21	0.076	Aliphatic and aryl amine
Cystine	1.55	<0.0001	1.81	<0.0001	1.16	0.23	Amino acid
Aspartic acid	1.59	0.0002	1.52	0.0032	1.69	0.0011	Amino acid
Arabinose	1.65	<0.0001	1.61	<0.0001	1.71	<0.0001	Carbohydrate
p-hydroxybenzoic acid	1.77	<0.0001	1.89	<0.0001	1.58	0.0004	Aromatic acid
Glutamic acid	1.82	<0.0001	1.54	<0.0001	2.24	<0.0001	Amino acid
2-hydroxy-butyrate	1.83	<0.0001	1.59	<0.0001	2.18	0.00050	Hydroxy acid
Kynurenine	1.83	<0.0001	1.74	<0.0001	1.96	<0.0001	Amino acid
meso-erythritol	2.83	0.0030	3.01	0.0065	2.57	0.042	Alcohol and polyol
Lactitol	7.44	0.0316	1.16	0.53	16.9	0.0013	Alcohol and polyol

The 7 metabolites subjected to multivariate analysis using the stepwise variable selection method are listed in Table 3. Among these metabolites, arabinose, meso-erythritol, and lactitol are excluded, because they are frequently consumed in the diet. The concentration of each metabolite in the colorectal cancer patients with stage 0–4, stage 0–2, or stage 3–4 disease at the training set was compared with that detected in the healthy volunteers, and the fold induction was calculated. P values were evaluated using the Mann-Whitney U test, and p values of less than 0.05 were considered to indicate a significant difference.

**Table 4:** Biomarkers for detecting colorectal cancer selected by the multiple logistic regression model.

Biomarkers	Coefficient	Standard error	p value	Lower 95% CI	Upper 95% CI
(Intercept)	−8.32	1.539	<0.0001	−11.71	−5.621
2-hydroxy-butyrate	286.59	71.90	<0.0001	155.0	440.1
Aspartic acid	33.87	14.29	0.0178	7.390	63.85
Kynurenine	1634.96	569.3	0.0041	559.1	2.830E+03
Cystamine	78.78	26.82	0.0033	31.53	137.3

The 4 metabolites selected by multiple logistic regression analysis using the stepwise variable selection method. The results of the analysis are shown in Table 4. The 95% confidence interval (95% CI) for the AUC (0.9097) obtained from ROC analysis ranged from 0.8438 to 0.9495.

The prediction model is as follows:

p = 1/[1+e−{−8.32+286.59(2-hydroxybutyrate)+33.87(aspartic acid)+1634.96(kynurenine)+78.78(cystamine)}].

**Figure 2:**
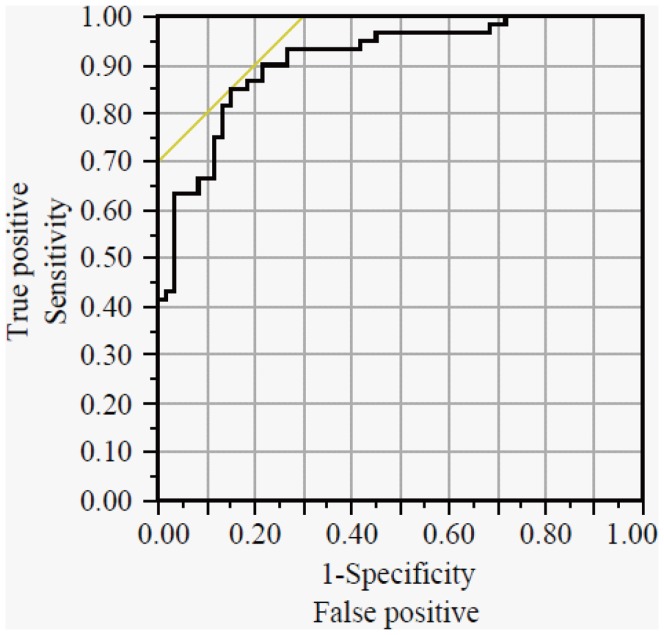
ROC curve of the established prediction model. The solid curve is the ROC curve for the prediction model established on the basis of the training set. The AUC and cut-off values were 0.9097 {95% confidence interval (95% CI): 0.8438–0.9495} and 0.4945, respectively. The sensitivity, specificity, and accuracy of this model are summarized in Table 5.

The Nagelkerke R^2^ value was 0.4533. The AUC, sensitivity, specificity, and accuracy of this model were 0.9097 {95% confidence interval (95% CI): from 0.8438 to 0.9495}, 85.0%, 85.0%, and 85.0%, respectively (Table 5, Figure 2). Although we selected 2, 3, 5, or 6 metabolites via the stepwise variable selection method and then performed further multiple logistic regression analyses, we could not establish a better model (data not shown). On the contrary, when the training set data were used, the sensitivity, specificity, and accuracy of CEA were 35.0%, 96.7%, and 65.8%, respectively, and those of CA19-9 were 16.7%, 100%, and 58.3%, respectively. Our prediction model also showed high sensitivity (83.3%) in the stage 0–2 colorectal cancer patient group, whereas CEA and CA19-9 displayed sensitivities of 30.6% and 5.6%, respectively.

**Table 5:** The sensitivity and specificity of tumor biomarkers and the prediction model.

		Training set							
	CEA			CA19-9			Prediction model		
	Stage 0–4	Stage 0–2	Stage 3–4	Stage 0–4	Stage 0–2	Stage 3–4	Stage 0–4	Stage 0–2	Stage 3–4
**Sensitivity**	35.0%	30.6%	37.5%	16.7%	5.6%	29.2%	85.0%	83.3%	87.5%
**Specificity**	96.7%	−	−	100%	−	−	85.0%	−	−
**Accuracy**	65.8%	−	−	58.3%	−	−	85.0%	−	−
									
		**Validation set**							
	**CEA**			**CA19-9**			**Prediction model**		
	**Stage 0–4**	**Stage 0–2**	**Stage 3–4**	**Stage 0–4**	**Stage 0–2**	**Stage 3–4**	**Stage 0–4**	**Stage 0–2**	**Stage 3–4**
**Sensitivity**	33.9%	6.9%	60.0%	13.6%	0%	26.7%	83.1%	82.8%	83.3%
**Specificity**	96.8%	−	−	100%	−	−	81.0%	−	−
**Accuracy**	66.4%	−	−	58.2%	−	−	82.0%	−	−

The sensitivity, specificity, and accuracy of CEA, CA19-9, and the prediction model were calculated using the cut-off value obtained from the ROC analysis. The cut-off values of CEA, CA19-9, and the prediction model were 5 ng/ml, 37 U/ml, and 0.4945, respectively.

The 27 metabolites selected in the training set (Table 2) and the established prediction model were examined using the validation set, which was composed of colorectal cancer patients (N = 59) and healthy volunteers (N = 63) (Table 1, Table S2). Regarding the 27 metabolites, the sensitivity, specificity, and accuracy of the training set were partially correlated with those of the validation set, and the correlation coefficients for these parameters were 0.425, 0.655, and 0.587, respectively (Figure S2). However, none of the metabolites displayed high sensitivity, specificity, and accuracy values (Table 2). On the contrary, when the validation set was used the sensitivity, specificity, and accuracy of the prediction model were 83.1%, 81.0%, and 82.0%, respectively, and these values were almost the same as those obtained with the training set (Table 5). In addition, the model also displayed high sensitivity for detecting stage 0–2 colorectal cancer (82.8%).

## Discussion

In this study, we investigated a new screening method for the early diagnosis of colorectal cancer based on GC/MS metabolomics. Although the training set included patients with early stage colorectal cancer, such as stage 0 or stage 1, the prediction model displayed high AUC (0.9097), sensitivity (85.0%), and accuracy (85.0%) values, which were higher than those of serum tumor markers (CA19-9 and CEA). In addition, when the validation set was used the model also exhibited high sensitivity for early stage colorectal cancer (83.1%). Taken together, the pathogenesis of colorectal cancer seems to lead to alterations in the levels of a variety of serum metabolites, although these fluctuations range from small to large.

Evaluations of data obtained by metabolomics should be treated carefully. For example, among the serum metabolites detected by our GC/MS-based metabolomic system, intra-day and inter-day variances were observed. For example, the tryptophan concentrations observed in the afternoon and at night were significantly decreased in comparison with those detected in the morning (Table S3 and Figure S3), and these differences might have been due to the effects of diet and/or daily activity. Previous studies have also demonstrated intra-day and inter-day variance in the levels of some amino acids [10], [11], [12]. Therefore it is important to evaluate the inter-day and intra-day variances of serum metabolite levels in metabolomic research, and blood samples collected before breakfast were used in this study. In addition, the accuracy of data obtained by instrumental analysis of serum samples should be carefully assessed because it can be affected by the method employed. Therefore, to discover reliable novel metabolite biomarkers, we calculated the RSD% values for the serum metabolite levels obtained by GC/MS analysis and then evaluated the inter-day and intra-day variances in the concentration of each serum metabolite. In GC/MS analysis, multiple peaks are sometimes detected for a particular metabolite. For example, there are variations in the number of TMS molecules that bind to certain molecules, and hence, major and minor peaks are detected. The minor peaks can be unstable, which can lead to incorrect interpretation of the data. Actually, in this study the RSD% values obtained by omitting the results for the minor peak-derived metabolites were better than those obtained using all of the data: when all data were used, the percentage of metabolites with RSD% values of less than 20% and 30% was 68.5% and 86.5%, respectively. In contrast, when the results for the minor peak-derived metabolites were omitted, they were 72.0% and 90.0%, respectively. Thus, the accurate evaluation of data obtained by metabolomics is expected to produce useful information. Based on these evaluations, 27 metabolites were selected as metabolite biomarker candidates, but these metabolites displayed individual AUC values of 0.6–0.8 and relatively low sensitivity or specificity (Table 2), indicating that single metabolite biomarkers are not practical for disease screening and/or diagnosis and that the use of multiple biomarkers might be better for discovering candidates with high sensitivity and specificity, although metabolomic approaches have been widely utilized to discover single disease biomarkers. Then, with the aim of evaluating the utility of multiple biomarkers for diagnosing colorectal cancer, multiple logistic regression analysis using the stepwise variable selection method (in this study) and principal component analysis (PCA) (data not shown) were carried out. However, PCA did not produce valuable results. PCA is an analytical method for analyzing a reduced number of variables; i.e., it is a dimension reduction technique, and has no dependent variables. On the contrary, multiple logistic regression analysis with the stepwise variable selection method can be used to select the optimal subset of variables and requires dependent variables. Thus, PCA might be unsuitable for large-scale studies because it can produce unexpected differences between the groups being compared, although supervised PCA, partial least squares discriminant analysis (PLS-DA), and orthogonal PLS-DA (OPLS-DA) may be applicable to establish the diagnosis models, because discrimination between colorectal cancer patients and controls was shown via OPLS-DA [13].

The established prediction model was composed of 4 metabolites; i.e., 2-hydroxybutyrate, aspartic acid, kynurenine, and cystamine (cysteamine+cystamine). 2-hydroxybutyrate is formed as a by-product during the formation of α-ketobutyrate via a reaction catalyzed by lactate dehydrogenase or α-hydroxybutyrate dehydrogenase. In a previous study [13], the increased level of 2-hydroxybutyric acid in sera of colorectal cancer patients was observed, being consistent with our results. The serum α-hydroxybutyrate dehydrogenase and total lactate dehydrogenase activities of ovarian cancer patients were significantly higher than those of patients with benign ovarian tumor [14], but the level of lactic acid in colorectal cancer was lower than that in healthy volunteers (Table S4). Therefore, 2-hydroxybutyrate would not be produced in the blood by lactate dehydrogenase, but the enzymatic production of 2-hydroxybutyrate by α-hydroxybutyrate dehydrogenase might be caused in blood and/or 2-hydroxybutyrate would be secreted from cells. Recently, it was demonstrated that 2-hydroxybutyrate is an early marker of both insulin resistance and impaired glucose regulation, and it was suggested that the underlying biochemical mechanisms might involve increased lipid oxidation and oxidative stress [15]. Direct and/or indirect actions of colorectal tumors might lead to upregulated levels of 2-hydroxybutyrate, although the mechanisms by which 2-hydroxybutyrate is regulated/converted in colorectal tissues remain unknown yet. Aspartic acid is basically produced from oxaloacetate by transamination. Increased serum/plasma levels of aspartic acid are observed in patients suffering from acute seizures [16] or Alzheimer’s disease [17]. A relationship between amino acid levels and cancer has recently been demonstrated by a large-scale study. The alterations in the serum levels of amino acids observed in cancer patients might be closely associated with the nutritional demands of tumor cells; i.e., tumor progression requires increased nutrient uptake. Regarding histidine, tyrosine and cysteine (cysteine+cystine), their serum levels in the colorectal cancer patients with stage 0–2 disease were higher than those with stage 3–4 disease, (Table S5). The serum level of lysine was significantly lower in the stage 3–4 colorectal cancer patients compared with the healthy volunteers. On the contrary, the serum levels of some amino acids including aspartic acid were more strongly enhanced in the stage 3–4 colorectal cancer patients compared with the stage 0–2 colorectal cancer patients (Table S5). In a previous study, various amino acids including aspartic acid displayed higher levels in colorectal tumor tissues than in normal colorectal tissues [5], [18], [19], indicating that tumor cells may need the nutrient. However, some inconsistencies among various research groups could be confirmed. Miyagi et al. and Qui et al. demonstrated that serum levels of most amino acids were decreased in the colorectal cancer patients [13], [20]. In the study by Bertini et al., serum levels of some amino acids were higher in the colorectal cancer patients, and others were lower [21]. However, in their reports, the results of aspartic acid were not represented, and therefore the relationship between amino acids and disease states of colorectal cancer need to be evaluated in detail. In addition, as suggested by Kimura et al. [22], it might be necessary to evaluate amino acid-to-amino acid, amino acid-to-other metabolite, amino acid-to protein, and/or amino acid-to-gene interaction networks in order to elucidate the mechanisms responsible for these phenomena. Kynurenine, which is a central compound in the tryptophan metabolism pathway, is used in the production of niacin. Previously, Huang et al. reported that the serum kynurenine/tryptophan ratio was higher in colorectal cancer patients than in ‘non-cancer’ controls, although no increased level of kynurenine was observed [23]. The ‘non-cancer’ controls did not seem to be healthy volunteers because they were also referred to as the ‘control patients’. Recently, higher levels of kynurenine were observed in samples from patients diagnosed with colon carcinoma, adenoma tubule villosum, or tubular adenoma than in those from the control group [24]. Tryptophan is metabolized to kynurenine by indoleamine 2,3-dioxygenase. Therefore, tryptophan metabolism might be upregulated in colorectal cancer patients. Cysteamine is the simplest stable aminothiol found in the body and is a product of the constitutive degradation of coenzyme A. Cysteamine is a precursor for the formation of hypotaurine (which is subsequently oxidized to taurine) by cysteamine dioxygenase. In this study, the colorectal cancer patients displayed significantly higher taurine serum levels than the healthy volunteers (Table S5). Cysteamine is also a degradation product of the amino acid cysteine. In our study, the colorectal cancer patients displayed significantly-higher cystine (cysteine+cystine) levels than the healthy volunteers (Table S5). In addition, the colorectal cancer patients with stage 0–2 disease demonstrated the higher levels of cystine (cysteine+cystine), cystamine (cysteamine+cystamine), and taurine than the colorectal cancer patients with stage 3–4 disease (Table S5), suggesting that tumor invasion and metastasis affect these metabolite levels. The higher levels of cysteine, taurine, and hypotaurine were observed in the colorectal cancer tissues compared with the normal tissue [5], [18]. Taken together, the metabolic pathways involving cysteine, cysteamine, and taurine might be useful for differentiating between colorectal cancer patients and healthy volunteers.

In conclusion, our findings suggest that GC/MS-based serum metabolomics could be used as a novel method for colorectal cancer screening tests. Research on new screening methods for the early diagnosis of colorectal cancer has been performed around the world, and the methylation of serum NEUROG1 [25], the serum dermokine level [26], and serum hydroxylated polyunsaturated ultra-long-chain fatty acid levels [27] have been demonstrated to be serum biomarker candidates for the early detection of colorectal cancer. We developed a metabolomics-based prediction model for colorectal cancer involving multiple biomarkers, and the sensitivity of the model for detecting early stage colorectal cancer patients was the same or better than those of previously described methods [25], [26], [27]. In this study, serum metabolome analysis was able to describe the status of colorectal cancer patients rather than simply detect the presence of colorectal cancer, which might be explained by our use of multiple biomarkers. Recently, the Human Serum Metabolome Consortium recommended methods for sample collection, sample preparation, and data acquisition for LC/MS and GC/MS in long-term and large-scale metabolomic studies [28], and metabolomic studies in the clinical research field have gradually been gaining attention. In metabolomics, information about various metabolites can be obtained via a single measurement. Determining the concentrations of the 4 metabolites selected in this study is crucial for the future clinical application of our model, and moreover the development of easier methods, for example enzyme-linked immunosorbent assay (ELISA) systems and procedures based on enzyme chemistry, is also important. Taken together, our findings will hopefully lead to an improved quality of life via the early detection of colorectal cancer.

## Supporting Information

Figure S1 **ROC curve of the metabolites that displayed significantly-different concentrations between the colorectal cancer patients and healthy volunteers.** The solid curve is the ROC curve for pyruvate+oxalacetic acid, 2-hydroxybutyrate, phosphate, isoleucine, nonanoic acid(C9), β-alanine, meso-erythritol, aspartic acid, pyroglutamic acid, creatinine, glutamic acid, p-hydroxybenzoic acid, arabinose, ribulose, asparagine, xylitol, O-phosphoethanolamine, ornithine, citrulline, glucuronate_1, glucosamine_2, palmitoleate, inositol, kynurenine, cysteamine+cystamine, cysteine+cystine, and lactitol obtained from the training set. The AUC, cut-off value, sensitivity, specificity, and accuracy values are summarized in Table 2.
(TIFF)

Figure S2 **The associations between the sensitivity, specificity, and accuracy values of the training and validation sets.** Regarding the 27 targeted metabolites, scatter plots of the sensitivity, specificity, and accuracy of the training and validation sets were produced, and then the associations between the training and validation sets were evaluated. The coefficients of correlation for the sensitivity, specificity, and accuracy values of the two sets were 0.425, 0.655, and 0.587, respectively.
(TIFF)

Figure S3 **The inter-day and intra-day variances of tryptophan.** The inter-day and intra-day variances of the serum levels of tryptophan were evaluated. To confirm the intra-day variance, blood was collected before breakfast (A), before lunch (B), and before dinner (C). For the inter-day variance, blood was collected before breakfast for a total of 3 days (Day 1, Day 2, and Day 3). The data are shown as mean ± standard deviation values (N = 16). Asterisks indicate the significant differences by the Wilcoxon signed-rank test and/or Steel-Dwass test (p<0.05).
(TIFF)

Table S1 **Subject information for the training set.**
(DOC)

Table S2 **Subject information for the validation set.**
(DOC)

Table S3 **The RSD%, inter-day variance, and intra-day variance values of serum metabolites.**
(DOC)

Table S4 **Comparison of serum metabolite levels between the colorectal cancer patients and healthy volunteers in the training set.**
(DOC)

Table S5 **Comparison of serum metabolite levels between the colorectal cancer patients and healthy volunteers in the data set mixing the training set with the validation set.**
(DOC)
