# Supplementary figures and images for: A Novel Serum Metabolomics-Based Diagnostic Approach for Colorectal Cancer
Source: PLoS One. 2012 Jul 11;7(7):e40459. doi: 10.1371/journal.pone.0040459 (PMC3394708; doi:10.1371/journal.pone.0040459)

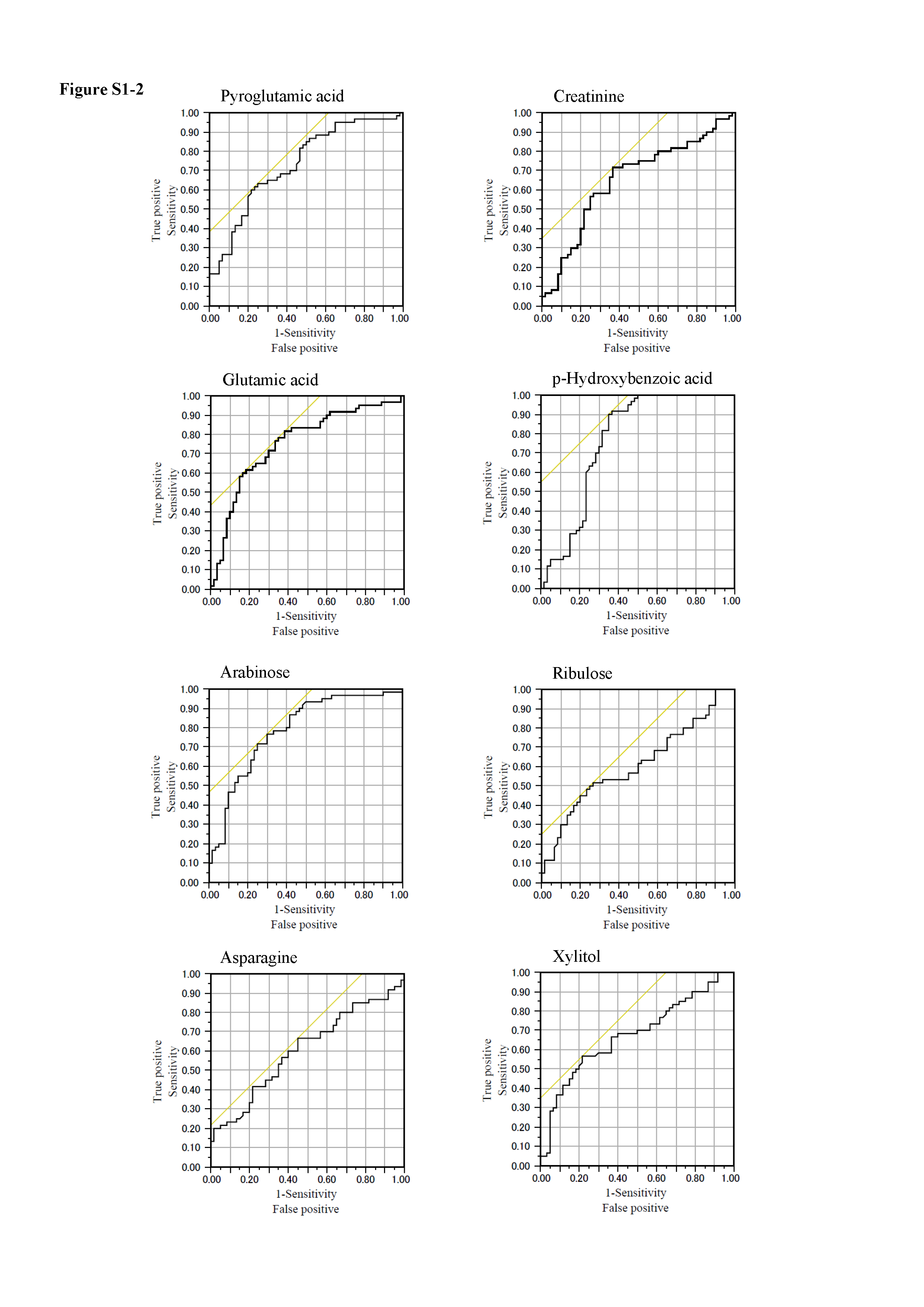

Supplement: Figure S1 — ROC curve of the metabolites that displayed significantly-different concentrations between the colorectal cancer patients and healthy volunteers. The solid curve is the ROC curve for pyruvate+oxalacetic acid, 2-hydroxybutyrate, phosphate, isoleucine, nonanoic acid(C9), β-alanine, meso-erythritol, aspartic acid, pyroglutamic acid, creatinine, glutamic acid, p-hydroxybenzoic acid, arabinose, ribulose, asparagine, xylitol, O-phosphoethanolamine, ornithine, citrulline, glucuronate_1, glucosamine_2, palmitoleate, inositol, kynurenine, cysteamine+cystamine, cysteine+cystine, and lactitol obtained from the training set. The AUC, cut-off value, sensitivity, specificity, and accuracy values are summarized in Table 2. (TIFF) [file pone.0040459.s001.tiff]

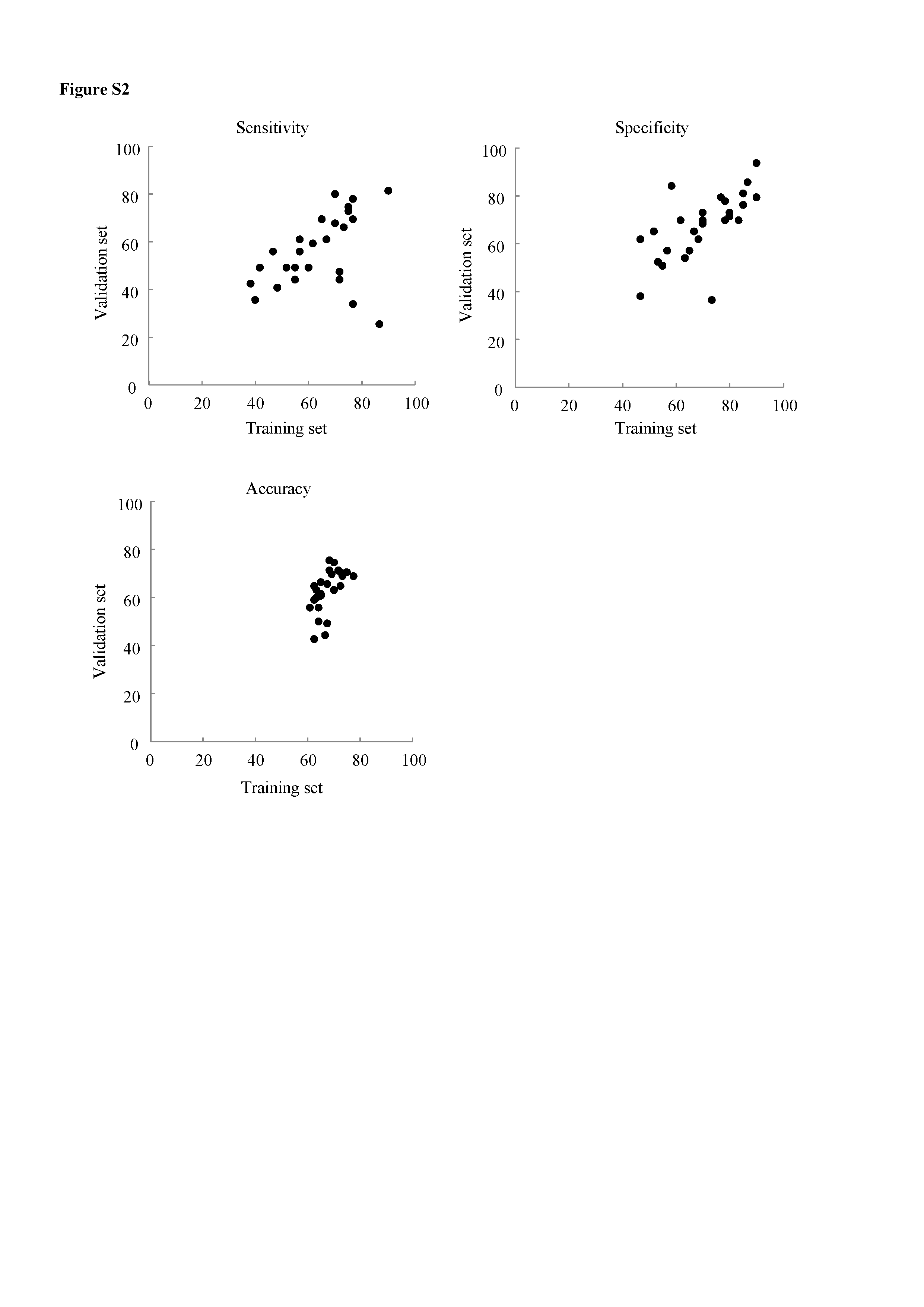

Supplement: Figure S2 — The associations between the sensitivity, specificity, and accuracy values of the training and validation sets. Regarding the 27 targeted metabolites, scatter plots of the sensitivity, specificity, and accuracy of the training and validation sets were produced, and then the associations between the training and validation sets were evaluated. The coefficients of correlation for the sensitivity, specificity, and accuracy values of the two sets were 0.425, 0.655, and 0.587, respectively. (TIFF) [file pone.0040459.s002.tiff]

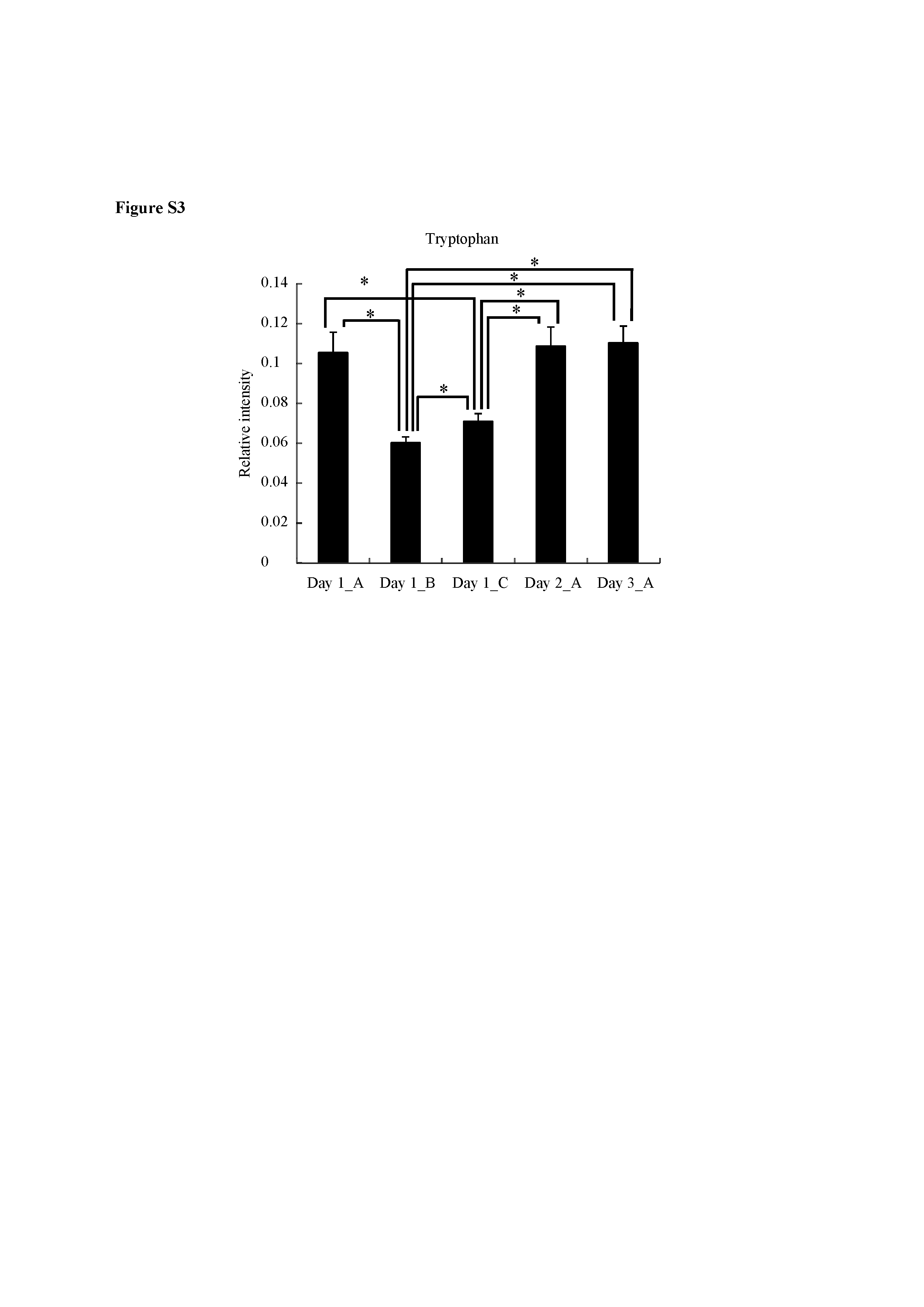

Supplement: Figure S3 — The inter-day and intra-day variances of tryptophan. The inter-day and intra-day variances of the serum levels of tryptophan were evaluated. To confirm the intra-day variance, blood was collected before breakfast (A), before lunch (B), and before dinner (C). For the inter-day variance, blood was collected before breakfast for a total of 3 days (Day 1, Day 2, and Day 3). The data are shown as mean ± standard deviation values (N = 16). Asterisks indicate the significant differences by the Wilcoxon signed-rank test and/or Steel-Dwass test (p<0.05). (TIFF) [file pone.0040459.s003.tiff]
