# Supplementary material for: A Novel Serum Metabolomics-Based Diagnostic Approach for Colorectal Cancer
Source: PLoS One. 2012 Jul 11;7(7):e40459. doi: 10.1371/journal.pone.0040459 (PMC3394708; doi:10.1371/journal.pone.0040459)
Supplement: Table S1 — Subject information for the training set. (DOC) [file pone.0040459.s004.doc]

**Table S1.** Subject information for the training set

| **No.** | **Age** | **Sex (M/F)** | **Stage** | **Tumor location** | **CEA (ng/ml)** | **CA19-9 (U/ml)** | **BMI (%)** |
| --- | --- | --- | --- | --- | --- | --- | --- |
| **Colorectal cancer patients** | | | | | | | |
| **1** | 59 | F | 0 | Rectum | 1.0 | 4.83 | 24.1 |
| **2** | 79 | M | 1 | Sigmoid colon | 2.5 | 9.0 | 22.1 |
| **3** | 74 | M | 0 | Ascending colon | 5.2 | 3.69 | 17.4 |
| **4** | 72 | M | 0 | Sigmoid colon | 1.6 | 8.0 | 23.6 |
| **5** | 51 | F | 0 | Rectum | 4.83 | 15.69 | 21.2 |
| **6** | 77 | F | 0 | Cecum | 3.1 | 24.6 | 20.4 |
| **7** | 81 | M | 0 | Transverse colon | 6.48 | 17.44 | 22.5 |
| **8** | 88 | M | 0 | Transverse colon | 2.2 | 13 | 18.4 |
| **9** | 70 | M | 0 | Ascending colon | 3.11 | <2.0 | 26.6 |
| **10** | 68 | M | 0 | Transverse colon | 5.68 | 24.09 | 26.9 |
| **11** | 75 | F | 0 | Ascending colon | 2.9 | 6.26 | 17.3 |
| **12** | 73 | F | 0 | Sigmoid colon | 1.25 | 11.98 | 24.4 |
| **13** | 58 | M | 4 | Sigmoid colon | 2119.6 | 85 | 20.4 |
| **14** | 58 | M | 4 | Rectum | 1013.2 | 551 | 23.3 |
| **15** | 62 | M | 4 | Sigmoid colon | 5.4 | 64 | 21.3 |
| **16** | 87 | F | 2 | Transverse colon | 22.5 | 5.0 | 23.1 |
| **17** | 47 | F | 4 | Transverse colon | 69.3 | 834 | 20.8 |
| **18** | 64 | M | 1 | Transverse colon | 1.4 | 8.0 | 18.2 |
| **19** | 48 | M | 2 | Cecum | 2.5 | 17 | 26.1 |
| **20** | 76 | M | 3 | Cecum | 2.7 | 8.0 | 26.0 |
| **21** | 83 | M | 1 | Rectum | 3.9 | 10 | 26.0 |
| **22** | 70 | F | 2 | Transverse colon | 1.0 | 3.0 | 22.8 |
| **23** | 70 | F | 2 | Sigmoid colon | 636.7 | 195 | 21.7 |
| **24** | 70 | M | 2 | Sigmoid colon | 14.7 | 12 | 25.7 |
| **25** | 66 | M | 4 | Sigmoid colon | 58 | 9.0 | 26.4 |
| **26** | 82 | M | 4 | Rectum | 1.5 | 10 | 19.9 |
| **27** | 55 | M | 4 | Rectum | 3.0 | 3.0 | 24.4 |
| **28** | 51 | M | 3 | Rectum | 1.2 | 17 | 20.2 |
| **29** | 83 | M | 2 | Sigmoid colon | 8.3 | 5.0 | 20.8 |
| **30** | 36 | F | 2 | Rectum | 1.0 | 4.0 | 22.3 |
| **31** | 60 | M | 2 | Rectum | 39.2 | 18 | 20.3 |
| **32** | 61 | M | 1 | Rectum | 10 | 7.9 | 22.3 |
| **33** | 45 | M | 4 | Descending colon | 2.8 | 16 | 24.3 |
| **34** | 73 | M | 4 | Sigmoid colon | 1.0 | 2.0 | 22.1 |
| **35** | 73 | M | 2 | Ascending colon | 3.4 | 10 | 23.9 |
| **36** | 48 | M | 3 | Rectum | 87.5 | 2.0 | 20.5 |
| **37** | 73 | M | 1 | Rectum | 3.6 | 8.0 | 21.6 |
| **38** | 66 | M | 3 | Sigmoid colon | 3.1 | 10 | 22.4 |
| **39** | 68 | M | 3 | Rectum | 5.9 | 9.0 | 20.3 |
| **40** | 71 | M | 3 | Rectum | 2.3 | 3.0 | 25.3 |
| **41** | 85 | M | 1 | Sigmoid colon | 0.6 | 48 | 19.2 |
| **42** | 73 | F | 1 | Sigmoid colon | 14.7 | 25 | 20.9 |
| **43** | 78 | F | 3 | Transverse colon | 1.5 | 4.0 | 23.6 |
| **44** | 60 | M | 4 | Rectum | 1.1 | 3.0 | 26.9 |
| **45** | 78 | F | 3 | Descending colon | 3.0 | 12 | 21.8 |
| **46** | 55 | M | 3 | Rectum | 4.0 | 33 | 19.8 |
| **47** | 85 | F | 4 | Transverse colon | 6.7 | 68 | 20.1 |
| **48** | 56 | F | 2 | Cecum | 3.6 | 9.0 | 18.6 |
| **49** | 83 | M | 3 | Rectum | 117.5 | 498 | 16.4 |
| **50** | 78 | F | 1 | Rectum | 2.8 | 9.0 | 17.3 |
| **51** | 68 | F | 1 | Rectum | 0.9 | 2.0 | 19.4 |
| **52** | 86 | M | 4 | Sigmoid colon | 4.3 | 52 | 21.4 |
| **53** | 79 | M | 3 | Cecum | 1.2 | <1.0 | 19.4 |
| **54** | 78 | F | 3 | Ascending colon | 1.5 | 6.0 | 27.1 |
| **55** | 75 | F | 2 | Ascending colon | 4.51 | 36.44 | 21.8 |
| **56** | 38 | M | 2 | Rectum | 3.4 | 54 | 20.7 |
| **57** | 43 | F | 1 | Ascending colon | 0.8 | 7.0 | 24.7 |
| **58** | 77 | M | 0 | Ascending colon | 11.8 | 12 | 19.8 |
| **59** | 58 | F | 1 | Ascending colon | 5.6 | 8.0 | 20.6 |
| **60** | 57 | M | 1 | Rectum | 2.3 | 8.0 | 20.0 |
|  |  |  |  |  |  |  |  |
| **Healthy volunteers** | | | | | | | |
| **1** | 73 | F | − | − | 1.32 | <2.0 | 21.0 |
| **2** | 76 | F | − | − | 4.7 | 15.96 | 23.4 |
| **3** | 73 | F | − | − | 1.82 | 9.04 | 22.5 |
| **4** | 68 | F | − | − | 1.4 | 12.9 | 16.6 |
| **5** | 65 | F | − | − | 0.97 | 7.78 | 19.7 |
| **6** | 76 | F | − | − | 2.59 | 13.84 | 22.5 |
| **7** | 68 | M | − | − | 4.26 | <2.0 | 21.1 |
| **8** | 78 | M | − | − | 7.49 | 14.55 | 20.9 |
| **9** | 73 | M | − | − | 11.3 | <2.0 | 26.0 |
| **10** | 69 | M | − | − | 4.9 | 3.89 | 22.5 |
| **11** | 70 | M | − | − | 3.98 | 4.6 | 20.2 |
| **12** | 54 | M | − | − | 2.3 | 2.9 | 25.3 |
| **13** | 78 | M | − | − | 1.1 | 10 | 21.5 |
| **14** | 70 | M | − | − | 2.9 | <2.0 | 22.7 |
| **15** | 60 | F | − | − | 2.1 | 3.8 | 31.6 |
| **16** | 52 | M | − | − | 2.9 | <2.0 | 25.9 |
| **17** | 83 | F | − | − | 2.05 | 4.47 | 19.5 |
| **18** | 56 | M | − | − | 4.0 | 3.74 | 23.7 |
| **19** | 64 | F | − | − | 1.52 | 3.79 | 20.3 |
| **20** | 60 | F | − | − | 1.4 | <2.0 | 18.7 |
| **21** | 88 | F | − | − | 7.6 | 20.14 | 20.9 |
| **22** | 47 | M | − | − | 1.94 | 7.06 | 23.0 |
| **23** | 51 | M | − | − | 1.5 | 7.2 | 22.3 |
| **24** | 48 | M | − | − | 1.6 | 7.9 | 24.2 |
| **25** | 47 | M | − | − | 0.7 | 4.7 | 22.7 |
| **26** | 72 | M | − | − | 4.3 | 2.0 | 21.2 |
| **27** | 66 | F | − | − | 2.9 | 23.9 | 20.7 |
| **28** | 39 | F | − | − | 1.02 | 11.38 | 19.0 |
| **29** | 69 | M | − | − | 3.1 | 6.4 | 18.8 |
| **30** | 74 | F | − | − | 2.2 | 12.9 | 18.3 |
| **31** | 74 | F | − | − | 5.0 | <2.0 | 18.9 |
| **32** | 50 | M | − | − | <0.5 | 2.54 | 20.8 |
| **33** | 60 | M | − | − | 1.28 | <2.0 | 18.6 |
| **34** | 79 | F | − | − | 3.13 | 3.68 | 18.2 |
| **35** | 54 | M | − | − | 4.6 | <2.0 | 26.7 |
| **36** | 53 | M | − | − | 1.5 | 8.7 | 23.6 |
| **37** | 71 | M | − | − | 2.8 | 9.6 | 24.3 |
| **38** | 62 | M | − | − | 4.2 | 18.2 | 24.8 |
| **39** | 71 | M | − | − | 1.6 | 18.2 | 22.7 |
| **40** | 72 | M | − | − | 0.9 | 13.3 | 28.6 |
| **41** | 50 | M | − | − | 2.8 | 16.8 | 25.2 |
| **42** | 73 | F | − | − | 1.0 | 12.4 | 24.7 |
| **43** | 55 | M | − | − | 1.2 | 9.3 | 23.5 |
| **44** | 71 | F | − | − | 1.9 | 3.2 | 22.0 |
| **45** | 53 | M | − | − | 1.0 | 5.9 | 16.4 |
| **46** | 60 | M | − | − | 2.4 | 5.7 | 21.8 |
| **47** | 69 | M | − | − | 1.5 | 8.4 | 23.5 |
| **48** | 50 | M | − | − | 1.1 | 6.2 | 28.4 |
| **49** | 60 | M | − | − | 0.7 | 2.3 | 20.4 |
| **50** | 70 | F | − | − | 3.6 | 19.9 | 20.0 |
| **51** | 60 | M | − | − | 2.3 | 18 | 21.9 |
| **52** | 72 | M | − | − | 3.6 | <2.0 | 20.3 |
| **53** | 75 | F | − | − | 2.2 | 7.7 | 21.0 |
| **54** | 74 | F | − | − | 2.2 | 22.8 | 20.2 |
| **55** | 70 | M | − | − | 1.8 | 7.2 | 21.6 |
| **56** | 60 | M | − | − | 2.1 | 16.5 | 18.1 |
| **57** | 76 | M | − | − | 2.2 | 24.1 | 22.6 |
| **58** | 53 | M | − | − | 2.4 | 16.8 | 20.7 |
| **59** | 50 | M | − | − | 1.3 | 4.5 | 25.2 |
| **60** | 55 | M | − | − | 1.5 | 6.8 | 22.1 |
