# Supplementary material for: A Novel Serum Metabolomics-Based Diagnostic Approach for Colorectal Cancer
Source: PLoS One. 2012 Jul 11;7(7):e40459. doi: 10.1371/journal.pone.0040459 (PMC3394708; doi:10.1371/journal.pone.0040459)
Supplement: Table S2 — Subject information for the validation set. (DOC) [file pone.0040459.s005.doc]

**Table S2. Subject information for the validation set**

| **No.** | **Age** | **Sex (M/F)** | **Stage** | **Tumor location** | **CEA (ng/ml)** | **CA19-9 (U/ml)** | **BMI (%)** |
| --- | --- | --- | --- | --- | --- | --- | --- |
| **Colorectal cancer patients** | | | | | | | |
| **1** | 69 | M | 0 | Rectum | 1.3 | 2.0 | 24.8 |
| **2** | 59 | M | 0 | Transverse colon | 6.26 | 27.92 | 25.3 |
| **3** | 62 | M | 1 | Sigmoid colon | 3.35 | 9.85 | 23.5 |
| **4** | 69 | F | 0 | Cecum | 0.65 | 5.8 | 19.8 |
| **5** | 66 | F | 4 | Sigmoid colon | 4362.8 | 7383 | 21.9 |
| **6** | 68 | M | 3 | Cecum | 1.6 | 4.0 | 27.9 |
| **7** | 60 | M | 4 | Sigmoid colon | 54.8 | 28 | 21.1 |
| **8** | 64 | F | 3 | Rectum | 2.5 | 6.0 | 24.3 |
| **9** | 68 | F | 4 | Rectum | 26.4 | 28 | 22.6 |
| **10** | 75 | M | 4 | Rectum | 52 | 19 | 21.8 |
| **11** | 67 | M | 0 | Ascending colon | 4.03 | <2.0 | 20.8 |
| **12** | 61 | M | 0 | Cecum | 1.42 | 4.65 | 23.2 |
| **13** | 71 | F | 1 | Rectum | 3 | <2.0 | 22.6 |
| **14** | 62 | F | 0 | Rectum | 3.24 | 10.02 | 21.0 |
| **15** | 66 | F | 0 | Cecum | 2 | <2.0 | 30.7 |
| **16** | 64 | M | 0 | Ascending colon | 4.07 | <2.0 | 22.8 |
| **17** | 66 | F | 0 | Rectum | 4.8 | 17.71 | 21.3 |
| **18** | 68 | F | 1 | Transverse colon | 1.9 | 17.22 | 22.4 |
| **19** | 75 | M | 4 | Sigmoid colon | 6.6 | 36 | 22.2 |
| **20** | 55 | F | 4 | Sigmoid colon | 2.9 | 10 | 25.7 |
| **21** | 55 | M | 4 | Ascending colon | 515.3 | 126 | 23.0 |
| **22** | 50 | F | 4 | Transverse colon | 6.1 | 34 | 20.9 |
| **23** | 73 | F | 4 | Sigmoid colon | 3.6 | 12 | 23.7 |
| **24** | 76 | F | 4 | Sigmoid colon | 15 | 16 | 24.5 |
| **25** | 52 | M | 4 | Sigmoid colon | 3.7 | <2.0 | 19.0 |
| **26** | 69 | M | 4 | Descending colon | 4.5 | <2.0 | 21.6 |
| **27** | 71 | M | 3 | Rectum | 1.9 | 5.0 | 24.0 |
| **28** | 77 | M | 3 | Rectum | 3.4 | 110 | 24.0 |
| **29** | 67 | M | 4 | Descending colon | 1325.7 | 1529 | 19.7 |
| **30** | 59 | M | 3 | Ascending colon | 1.4 | 10 | 23.1 |
| **31** | 65 | M | 4 | Rectum | 89.1 | 16 | 24.8 |
| **32** | 63 | M | 3 | Sigmoid colon | 2.3 | <2.0 | 19.0 |
| **33** | 71 | F | 1 | Rectum | 5.1 | 8.0 | 19.8 |
| **34** | 63 | M | 4 | Rectum | 26.5 | 22 | 21.8 |
| **35** | 74 | M | 4 | Sigmoid colon | 22.8 | 62 | 20.0 |
| **36** | 60 | F | 1 | Ascending colon | 1.9 | 18 | 17.6 |
| **37** | 58 | F | 2 | Sigmoid colon | 2.7 | <2.0 | 18.9 |
| **38** | 73 | M | 1 | Cecum | 2 | 2.0 | 21.6 |
| **39** | 55 | M | 3 | Cecum | 12.9 | 23 | 28.0 |
| **40** | 61 | F | 1 | Ascending colon | 1.4 | 5.0 | 20.4 |
| **41** | 71 | F | 4 | Sigmoid colon | 3936 | 13611 | 25.2 |
| **42** | 72 | F | 4 | Sigmoid colon | 4350 | 587 | 21.9 |
| **43** | 67 | M | 3 | Descending colon | 2.6 | 4.0 | 22.3 |
| **44** | 71 | M | 1 | Sigmoid colon | 1.2 | 4.0 | 21.6 |
| **45** | 31 | F | 2 | Sigmoid colon | 1 | 31 | 18.2 |
| **46** | 74 | F | 2 | Ascending colon | 1.3 | 16 | 20.9 |
| **47** | 73 | M | 3 | Ascending colon | 3 | 8.0 | 23.7 |
| **48** | 65 | F | 1 | Sigmoid colon | 3.8 | 13 | 20.6 |
| **49** | 81 | M | 4 | Rectum | 19.2 | 13 | 22.8 |
| **50** | 51 | M | 3 | Transverse colon | 5.2 | 2.0 | 27.4 |
| **51** | 65 | F | 1 | Sigmoid colon | 2 | 2.6 | 26.2 |
| **52** | 84 | M | 3 | Sigmoid colon | 312 | 617 | 19.2 |
| **53** | 40 | M | 1 | Rectum | 0.8 | 9.0 | 19.6 |
| **54** | 68 | F | 0 | Cecum | 3.25 | <2.0 | 25.8 |
| **55** | 57 | F | 0 | Rectum | 1.89 | 7.49 | 22.4 |
| **56** | 58 | F | 0 | Rectum | 2.14 | 17.21 | 17.3 |
| **57** | 60 | F | 0 | Cecum | 4.59 | 23.52 | 21.0 |
| **58** | 61 | F | 0 | Cecum | 0.62 | 9.81 | 22.9 |
| **59** | 68 | F | 0 | Rectum | 2.43 | 7.0 | 26.0 |
|  |  |  |  |  |  |  |  |
| **Healthy volunteers** | | | | | | | |
| **1** | 64 | F | − | − | 1.02 | 4.79 | 20.1 |
| **2** | 60 | M | − | − | 1.84 | 10.92 | 22.4 |
| **3** | 56 | M | − | − | 3.19 | 10.04 | 22.2 |
| **4** | 60 | M | − | − | 3.43 | 2.58 | 22.2 |
| **5** | 62 | M | − | − | 0.5 | 14.62 | 22.0 |
| **6** | 67 | M | − | − | 0.8 | 2.9 | 20.1 |
| **7** | 61 | M | − | − | 1.87 | 12.35 | 21.9 |
| **8** | 48 | F | − | − | 1.09 | 10.23 | 22.2 |
| **9** | 70 | M | − | − | 3 | 8.1 | 20.6 |
| **10** | 61 | M | − | − | 1.25 | 7.8 | 24.2 |
| **11** | 69 | M | − | − | 3.4 | 9.8 | 21.7 |
| **12** | 70 | M | − | − | 3.2 | 5.8 | 20.9 |
| **13** | 65 | F | − | − | 2.5 | 10.6 | 24.0 |
| **14** | 67 | M | − | − | 2.2 | 19.3 | 21.8 |
| **15** | 67 | M | − | − | 2.32 | 2.02 | 30.1 |
| **16** | 60 | F | − | − | 1.36 | 4.32 | 23.7 |
| **17** | 64 | F | − | − | 2.4 | 4.79 | 18.9 |
| **18** | 69 | F | − | − | 1.25 | <2.0 | 26.0 |
| **19** | 73 | F | − | − | 2.41 | 3.93 | 25.5 |
| **20** | 65 | F | − | − | 2.9 | 10.28 | 23.7 |
| **21** | 52 | M | − | − | 1.97 | 6.56 | 24.4 |
| **22** | 61 | F | − | − | 1.12 | 20.36 | 22.4 |
| **23** | 62 | F | − | − | 5.05 | 7.9 | 21.8 |
| **24** | 62 | F | − | − | 2.72 | 2.84 | 14.2 |
| **25** | 63 | F | − | − | 2.45 | 17.35 | 18.4 |
| **26** | 63 | F | − | − | 1.88 | <2.0 | 22.8 |
| **27** | 67 | M | − | − | 1.16 | <2.0 | 24.1 |
| **28** | 68 | M | − | − | 3.16 | 2.2 | 21.2 |
| **29** | 63 | M | − | − | 3.15 | 3.37 | 25.5 |
| **30** | 60 | M | − | − | 5.82 | 2.23 | 20.0 |
| **31** | 64 | M | − | − | 1.24 | 7.99 | 22.7 |
| **32** | 63 | M | − | − | 4.1 | 1.8 | 21.5 |
| **33** | 63 | M | − | − | 1.2 | 4.9 | 18.9 |
| **34** | 65 | M | − | − | 1.7 | 7.0 | 20.7 |
| **35** | 61 | F | − | − | 4.6 | <2.0 | 21.2 |
| **36** | 54 | M | − | − | 1.77 | 7.87 | 21.6 |
| **37** | 47 | M | − | − | 1.3 | 5.8 | 20.3 |
| **38** | 66 | F | − | − | 1.5 | 6.7 | 18.9 |
| **39** | 61 | M | − | − | 0.5 | 10.2 | 21.1 |
| **40** | 72 | F | − | − | <0.5 | 7.0 | 23.8 |
| **41** | 63 | F | − | − | 3.7 | 2.0 | 21.8 |
| **42** | 50 | M | − | − | 3.34 | 2.53 | 24.4 |
| **43** | 54 | M | − | − | 1.41 | 8.0 | 24.7 |
| **44** | 60 | F | − | − | 2.74 | <2.0 | 18.9 |
| **45** | 61 | F | − | − | 1.27 | 45.58 | 19.3 |
| **46** | 63 | F | − | − | 1.77 | <2.0 | 21.4 |
| **47** | 64 | F | − | − | 1.58 | <2.0 | 22.1 |
| **48** | 60 | M | − | − | 1.1 | 9.7 | 23.5 |
| **49** | 53 | M | − | − | 0.5 | 7.7 | 23.8 |
| **50** | 64 | F | − | − | 1.4 | 9.0 | 22.7 |
| **51** | 60 | F | − | − | 1.3 | 9.2 | 21.5 |
| **52** | 71 | F | − | − | 1.7 | 8.6 | 22.1 |
| **53** | 62 | F | − | − | 1.4 | 9.8 | 22.9 |
| **54** | 70 | F | − | − | 1.6 | 8.8 | 23.5 |
| **55** | 64 | M | − | − | 3.5 | 12.1 | 22.2 |
| **56** | 64 | F | − | − | 0.8 | 5.3 | 24.1 |
| **57** | 60 | F | − | − | 1.5 | 5.7 | 27.1 |
| **58** | 61 | M | − | − | 1 | 7.2 | 25.3 |
| **59** | 61 | M | − | − | 10.7 | 18 | 20.7 |
| **60** | 71 | F | − | − | 1.3 | 5.5 | 20.5 |
| **61** | 67 | F | − | − | 1.2 | 16.6 | 17.8 |
| **62** | 62 | F | − | − | 0.9 | 5.9 | 25.7 |
| **63** | 68 | M | − | − | 1.1 | 2.8 | 24.3 |
