# Supplementary material for: A Novel Serum Metabolomics-Based Diagnostic Approach for Colorectal Cancer
Source: PLoS One. 2012 Jul 11;7(7):e40459. doi: 10.1371/journal.pone.0040459 (PMC3394708; doi:10.1371/journal.pone.0040459)
Supplement: Table S3 — The RSD%, inter-day variance, and intra-day variance values of serum metabolites. (DOC) [file pone.0040459.s006.doc]

**Table S3. The RSD%, inter-day variance, and intra-day variance values of serum metabolites**

|  |  |  | **RSD%** |  | **Inter-day variance** | |  | **Intra-day variance** | | | | | |
| --- | --- | --- | --- | --- | --- | --- | --- | --- | --- | --- | --- | --- | --- |
|  |  |  |  |  | **Wilcoxon** | **Steel-**  **Dwass** |  | **Wilcoxon** | **Steel-**  **Dwass** | **Wilcoxon** | **Steel-**  **Dwass** | **Wilcoxon** | **Steel-**  **Dwass** |
| **RT** | **Q-ion** | **Compounds** |  |  |  |  |  | **Morning vs Daytime** | | **Morning vs Night** | | **Daytime vs Night** | |
| 280.05000 | 152 | 2-hydroxypyridine | Background |  |  |  |  |  |  |  |  |  |  |
| 285.10002 | 174 | Pyruvate+oxalacetic acid | 10.7 |  |  |  |  |  |  |  |  |  |  |
| 291.25002 | 147 | Lactic acid | 4.4 |  |  |  |  |  |  | 0.040 |  |  |  |
| 303.10002 | 205 | Glycolic acid | 10.4 |  |  |  |  |  |  |  |  |  |  |
| 306.60000 | 116 | N-methylethanolamine | Background |  |  |  |  |  |  |  |  |  |  |
| 321.70002 | 116 | Alanine(2TMS) | 6.5 |  |  |  |  |  |  |  |  |  |  |
| 326.45004 | 202 | n-butylamine | Background |  |  |  |  |  |  |  |  |  |  |
| 334.99998 | 204 | Glycine(2TMS) | 18.9 |  |  |  |  |  |  |  |  |  |  |
| 337.09998 | 205 | 2-hydroxy-butyrate | 14.0 |  |  |  |  |  |  |  |  |  |  |
| 340.10004 | 100 | Oxalate | 30.7 |  |  |  |  |  |  |  |  |  |  |
| 343.30002 | 202 | Ketovaline_2 | 16.1 |  |  |  |  |  |  |  |  |  |  |
| 345.79998 | 116 | Sarcosine | 47.4 |  |  |  |  |  |  |  |  |  |  |
| 359.85000 | 117 | 3-hydroxy-butyrate | 7.0 |  |  |  |  |  |  | 0.0365 |  | 0.0185 | 0.0485 |
| 367.99998 | 131 | 2-aminobutyric acid | 30.9 |  |  |  |  |  |  |  |  |  |  |
| 372.49998 | 200 | Ketoisoleucine_1 | 11.4 |  |  |  |  | 0.0136 | 0.0362 |  |  |  |  |
| 385.45002 | 96 | Ketoisoleucine_2 | 18.5 |  |  |  |  |  |  |  |  |  |  |
| 395.95002 | 144 | Valine(2TMS) | 8.8 |  |  |  |  | 0.0479 |  |  |  |  |  |
| 418.24998 | 116 | Dihydroxyacetone | 26.3 |  | 0.0044 | 0.0123 |  |  |  |  |  |  |  |
| 422.74998 | 116 | Serine(2TMS) | 10.8 |  |  |  |  |  |  |  |  |  |  |
| 428.05002 | 174 | 2-aminoethanol | 5.8 |  |  |  |  |  |  | 0.0079 | 0.0215 |  |  |
| 428.25000 | 201 | n-caprylic acid | 9.8 |  |  |  |  | 0.0302 |  |  |  | 0.040 |  |
| 428.80002 | 147 | Glycerol | 6.2 |  |  |  |  |  |  |  |  | 0.0019 | 0.0053 |
| 429.10002 | 299 | Phosphate | 9.9 |  |  |  |  |  |  |  |  |  |  |
| 429.94998 | 158 | Leucine | 14.9 |  |  |  |  |  |  |  |  |  |  |
| 443.25000 | 159 | Isoleucine | 11.6 |  |  |  |  |  |  |  |  |  |  |
| 444.79998 | 130 | Threonine(2TMS) | 10.1 |  |  |  |  |  |  |  |  |  |  |
| 448.39998 | 142 | Proline | 13.1 |  |  |  |  |  |  |  |  |  |  |
| 451.99998 | 174 | Glycine(3TMS) | 4.8 |  |  |  |  |  |  |  |  |  |  |
| 456.15000 | 247 | Succinic acid (or aldehyde) | 7.3 |  |  |  |  |  |  |  |  |  |  |
| 462.15000 | 292 | Glyceric acid | 30.2 |  |  |  |  |  |  |  |  |  |  |
| 476.80002 | 245 | Fumaric acid | 24.8 |  |  |  |  |  |  |  |  |  |  |
| 479.74998 | 204 | Serine(3TMS) | 20.5 |  |  |  |  |  |  |  |  |  |  |
| 483.19998 | 262 | Alanine(3TMS) | 10.2 |  |  |  |  |  |  |  |  |  |  |
| 484.40004 | 215 | Nonanoic acid(C9) | 14.0 |  |  |  |  |  |  |  |  |  |  |
| 494.00004 | 117 | Threonine(3TMS) | 18.3 |  |  |  |  |  |  |  |  |  |  |
| 506.10000 | 234 | 2,3-bisphospho-glycerate | 12.0 |  |  |  |  | 0.0011 | 0.0032 |  |  | 0.0167 | 0.0441 |
| 520.05000 | 248 | β-alanine | 9.5 |  |  |  |  |  |  |  |  |  |  |
| 520.35000 | 104 | Hydrocinnamate | 19.0 |  |  |  |  |  |  |  |  |  |  |
| 548.40000 | 133 | Malic acid | 35.2 |  |  |  |  |  |  |  |  |  |  |
| 551.65002 | 217 | Threitol | 20.3 |  |  |  |  |  |  |  |  |  |  |
| 555.94998 | 217 | meso-erythritol | 7.3 |  |  |  |  |  |  |  |  |  |  |
| 565.30002 | 267 | Acetylsalicylic acid | 8.6 |  |  |  |  |  |  | 0.0016 | 0.0047 | 0.0031 | 0.0087 |
| 565.45002 | 232 | Aspartic acid | 13.9 |  |  |  |  |  |  |  |  |  |  |
| 568.65000 | 176 | Methionine | 15.1 |  |  |  |  |  |  |  |  |  |  |
| 569.30004 | 140 | trans-4-hydroxy-L-proline | 16.5 |  |  |  |  |  |  |  |  |  |  |
| 571.54998 | 156 | Pyroglutamic acid | 15.7 |  |  |  |  |  |  |  |  |  |  |
| 579.04998 | 239 | Pyrogallol | 9.9 |  |  |  |  |  |  |  |  |  |  |
| 586.65000 | 329 | Creatinine | 14.8 |  |  |  |  |  |  |  |  |  |  |
| 594.00000 | 275 | 2-isopropylmalic acid | Internal standard |  |  |  |  |  |  |  |  |  |  |
| 611.50002 | 85 | β-glutamic acid | 20.6 |  |  |  |  |  |  |  |  |  |  |
| 614.44998 | 246 | Glutamic acid | 5.4 |  |  |  |  |  |  |  |  |  |  |
| 617.70000 | 103 | Anthranilic acid | 22.6 |  | 0.0365 |  |  |  |  | 0.0205 |  |  |  |
| 622.69998 | 218 | Phenylalanine | 6.1 |  |  |  |  | 0.0079 | 0.0215 |  |  |  |  |
| 623.40000 | 223 | p-hydroxybenzoic acid | 18.2 |  |  |  |  |  |  |  |  |  |  |
| 624.40002 | 217 | Lyxose_1 (or Xylose_1) | 43.0 |  |  |  |  |  |  |  |  |  |  |
| 627.40002 | 103 | Xylose_2 | 19.2 |  |  |  |  |  |  |  |  |  |  |
| 628.29996 | 179 | 4-hydroxyphenylacetic acid | 22.9 |  |  |  |  |  |  |  |  |  |  |
| 629.14998 | 117 | Lyxose_2 | 26.0 |  |  |  |  |  |  |  |  |  |  |
| 630.69996 | 103 | Threo-β-hydroxy aspartic acid | 12.1 |  |  |  |  |  |  | 0.0365 |  |  |  |
| 630.94998 | 217 | Arabinose | 16.2 |  |  |  |  |  |  |  |  |  |  |
| 633.64998 | 257 | Lauric acid | 11.0 |  |  |  |  |  |  | 0.0226 |  |  |  |
| 635.35002 | 98 | N-acetyl-L-aspartic acid_1 | 34.9 |  |  |  |  |  |  |  |  |  |  |
| 637.30002 | 89 | Ribulose | 18.2 |  |  |  |  |  |  |  |  |  |  |
| 637.85004 | 217 | Ribose | 35.2 |  |  |  |  |  |  |  |  |  |  |
| 638.80002 | 231 | Asparagine | 8.4 |  |  |  |  |  |  |  |  |  |  |
| 641.25000 | 326 | Taurine | 11.0 |  | 0.0226 |  |  |  |  |  |  |  |  |
| 650.25000 | 217 | Xylitol | 14.7 |  |  |  |  |  |  |  |  |  |  |
| 652.75002 | 147 | Phthalic acid | 21.9 |  |  |  |  |  |  |  |  |  |  |
| 655.89996 | 204 | 1,6-anhydroglucose | 20.9 |  |  |  |  |  |  |  |  |  |  |
| 656.80002 | 103 | Arabitol | 17.9 |  |  |  |  |  |  |  |  |  |  |
| 659.14998 | 217 | Ribitol | 67.0 |  |  |  |  |  |  |  |  |  |  |
| 661.40004 | 160 | Rhamnose_2 | 23.9 |  |  |  |  |  |  |  |  |  |  |
| 671.85000 | 103 | Putrescine | 28.0 |  | 0.0438 |  |  |  |  | 0.0365 |  |  |  |
| 672.85002 | 229 | Aconitate | 42.4 |  |  |  |  |  |  |  |  |  |  |
| 685.89996 | 156 | Glutamine | 7.4 |  |  |  |  |  |  |  |  |  |  |
| 687.19998 | 267 | 4-hydroxymandelate | 11.4 |  |  |  |  |  |  | 0.0027 | 0.0077 | 0.0056 | 0.0155 |
| 687.40002 | 209 | Methoxy-4-hydroxyphenylacetate | 15.4 |  |  |  |  |  |  | 0.011 | 0.0295 |  |  |
| 689.05002 | 100 | O-phosphoethanolamine | 18.3 |  |  |  |  |  |  |  |  |  |  |
| 697.14996 | 204 | Shikimic acid | Background |  |  |  |  |  |  |  |  |  |  |
| 701.85000 | 248 | Glycyl-glycine_1 | 19.0 |  |  |  |  |  |  |  |  |  |  |
| 702.30000 | 273 | Citric acid + isocitric acid | 5.0 |  |  |  |  |  |  | 0.0365 |  |  |  |
| 703.95000 | 200 | Ornithine | 4.0 |  |  |  |  |  |  |  |  |  |  |
| 705.40002 | 265 | Hypoxanthine | 11.8 |  |  |  |  | 0.0205 |  |  |  |  |  |
| 707.29998 | 157 | Citrulline | 12.6 |  |  |  |  |  |  |  |  |  |  |
| 711.90000 | 217 | Tagatose_1 | 101.8 |  |  |  |  |  |  |  |  |  |  |
| 714.15000 | 129 | Dimethylbenzimidazole | Background |  |  |  |  |  |  |  |  |  |  |
| 715.95000 | 217 | Psicose_1 | 43.4 |  |  |  |  |  |  |  |  |  |  |
| 718.20000 | 147 | 1,5-anhydro-D-glucitol | 1.8 |  |  |  |  |  |  |  |  |  |  |
| 720.40002 | 217 | Tagatose_2 (or Psicose_2) | 24.6 |  |  |  |  |  |  |  |  |  |  |
| 722.29998 | 200 | Lysine (3TMS) | 39.8 |  |  |  |  |  |  |  |  |  |  |
| 722.55000 | 217 | α-sorbopyranose_1 (or Fructose_1) | 10.1 |  |  |  |  | 0.0056 | 0.0155 | 0.0098 | 0.0266 |  |  |
| 723.75000 | 206 | Hippurate_2 | 23.6 |  |  |  |  |  |  |  |  |  |  |
| 726.55002 | 217 | Fructose_2 | 14.7 |  |  |  |  |  |  |  |  |  |  |
| 728.90004 | 147 | Mannose_1 | 2.8 |  |  |  |  |  |  | <0.0001 | 0.0002 | 0.0070 | 0.0193 |
| 729.79998 | 176 | 5-dehydroquinic acid | 13.5 |  |  |  |  |  |  | 0.0167 | 0.0441 |  |  |
| 733.80000 | 205 | Glucose_1 | 13.4 |  | 0.0332 |  |  |  |  |  |  |  |  |
| 736.54998 | 147 | Allose_2 | 43.4 |  |  |  |  |  |  |  |  |  |  |
| 737.55000 | 133 | Sebacic acid | 28.8 |  |  |  |  |  |  |  |  |  |  |
| 740.85000 | 205 | Galactose_2 | 149.8 |  |  |  |  |  |  |  |  |  |  |
| 742.65000 | 147 | Glucose_2 | 8.8 |  |  |  |  |  |  |  |  |  |  |
| 747.55002 | 217 | Gulcono-1,4-lactone | 51.7 |  |  |  |  |  |  |  |  |  |  |
| 747.65004 | 156 | Lysine(4TMS) | 2.2 |  |  |  |  |  |  |  |  |  |  |
| 749.20002 | 155 | Histidine | 6.9 |  |  |  |  |  |  |  |  |  |  |
| 749.80002 | 205 | Galactosamine_1 | 28.7 |  |  |  |  |  |  |  |  |  |  |
| 750.30000 | 217 | Glucuronate_1 | 10.8 |  |  |  |  |  |  |  |  |  |  |
| 752.10000 | 129 | Glucosamine_2 | 8.5 |  |  |  |  |  |  |  |  |  |  |
| 754.15002 | 205 | Galactosamine_2 | 28.4 |  |  |  |  |  |  |  |  |  |  |
| 756.30000 | 218 | Tyrosine | 4.7 |  |  |  |  | <0.0001 | 0.0001 |  |  |  |  |
| 757.30002 | 332 | Ascorbic acid | 20.1 |  |  |  |  |  |  |  |  |  |  |
| 757.75002 | 89 | Glucuronate_2 | 8.3 |  |  |  |  |  |  |  |  |  |  |
| 760.39998 | 281 | Gallic acid | 20.8 |  |  |  |  |  |  | 0.0019 | 0.0053 |  |  |
| 765.60000 | 299 | 1-hexadecanol | 14.0 |  |  |  |  | 0.0006 | 0.0019 | 0.0024 | 0.0068 |  |  |
| 766.70004 | 218 | Coniferyl aldehyde_2 | 15.2 |  |  |  |  |  |  |  |  |  |  |
| 767.34996 | 172 | N-α-acetyl-L-ornithine_1 | 29.8 |  |  |  |  |  |  |  |  |  |  |
| 775.20000 | 237 | Paraxanthine | 10.1 |  |  |  |  |  |  |  |  |  |  |
| 779.59998 | 149 | N-α-acetyl-L-ornithine_2 | 14.7 |  |  |  |  |  |  |  |  |  |  |
| 781.74996 | 218 | S-benzyl-L-cysteine_1 | 15.5 |  |  |  |  |  |  | 0.0079 | 0.00215 |  |  |
| 792.34998 | 95 | Palmitoleate | 18.3 |  |  |  |  |  |  |  |  |  |  |
| 810.94998 | 174 | Dopamine | 21.6 |  |  |  |  |  |  |  |  |  |  |
| 812.59998 | 147 | Inositol | 3.5 |  |  |  |  |  |  |  |  |  |  |
| 815.79996 | 315 | Arabinose-5-phosphate_2 | 9.3 |  |  |  |  |  |  |  |  |  |  |
| 816.25002 | 441 | Uric acid | 4.8 |  |  |  |  |  |  |  |  |  |  |
| 823.54998 | 174 | N-α-acetyl-L-lysine_2 | 73.9 |  |  |  |  |  |  |  |  |  |  |
| 836.70000 | 327 | Heptadecanoate | 18.0 |  |  |  |  |  |  | 0.0249 |  |  |  |
| 853.95000 | 218 | Kynurenine | 13.2 |  |  |  |  |  |  |  |  |  |  |
| 859.80000 | 100 | Cysteamine+cystamine | 5.6 |  |  |  |  |  |  |  |  |  |  |
| 864.00000 | 202 | Tryptophan | 8.7 |  |  |  |  | 0.0001 | 0.0003 | 0.0063 | 0.0173 | 0.0001 |  |
| 865.75002 | 95 | Elaidic acid | 20.7 |  |  |  |  |  |  |  |  |  |  |
| 887.34996 | 218 | Cysteine+cystine | 15.3 |  |  |  |  |  |  |  |  |  |  |
| 937.35000 | 129 | 2'-deoxyuridine_2 | 27.8 |  |  |  |  |  |  |  |  |  |  |
| 1025.50002 | 204 | Lactitol | 15.0 |  |  |  |  |  |  |  |  |  |  |

The metabolites detected in human serum along with their retention times (RT) and quantitative ions (Q-ion) are listed in Table S3. The stability of the serum concentrations of the metabolites assessed in this study was evaluated by calculating RSD% values. The term 'Background' indicates that the metabolites were extracted using non-standard methods. 2-isopropylmalic acid was used as an internal standard. The inter-day variance (for 3 days) and intra-day variance (morning, daytime, and night) values of the serum metabolites were also evaluated using the Wilcoxon signed-rank test and Steel-Dwass test, respectively. P values of less than 0.05 were considered to indicate a significant difference. The term 'Pyruvate+oxalacetic acid' shows that oxalacetic acid was converted to pyruvate during the pre-treatment procedure and so was detected as pyruvate by GC/MS. The term 'Citric acid + isocitric acid' shows that citric acid and isocitric acid were detected at the same RT. The term 'Cysteamine+cystamine' shows that cysteamine was converted to cystamine during the pre-treatment procedure and so was detected as cystamine by GC/MS. The term 'Cysteine+cystine' shows that cysteine was converted to cystine during the pre-treatment procedure and so was detected as cystine by GC/MS. In GC/MS analysis, multiple peaks are sometimes detected for a particular metabolite due to TMS-derivatization, isomeric form etc. In such cases, each metabolite had the term ‘_1’, ‘_2’, or ‘(-TMS)’ added to the end of its name, according to the method described in a previous report [7].
