# Supplementary material for: A Novel Serum Metabolomics-Based Diagnostic Approach for Colorectal Cancer
Source: PLoS One. 2012 Jul 11;7(7):e40459. doi: 10.1371/journal.pone.0040459 (PMC3394708; doi:10.1371/journal.pone.0040459)
Supplement: Table S4 — Comparison of serum metabolite levels between the colorectal cancer patients and healthy volunteers in the training set. (DOC) [file pone.0040459.s007.doc]

**Table S4.** Comparison of serum metabolite levels between the colorectal cancer patients and healthy volunteers in the training set

|  |  |  | **Healthy volunteers** | **Colorectal cancer patients** | | |  | **Colorectal cancer patients** | | | | | |
| --- | --- | --- | --- | --- | --- | --- | --- | --- | --- | --- | --- | --- | --- |
|  |  |  |  | **Stage 0-4** | **Stage 0-2** | **Stage 3-4** |  | **Stage 0-4** | | **Stage 0-2** | | **Stage 3-4** | |
| **RT** | **Q-ion** | **Compounds** |  |  |  |  |  | **Fold induction** | **P value** | **Fold induction** | **P value** | **Fold induction** | **P value** |
| 285.10002 | 174 | Pyruvate+oxalacetic acid | 0.0325 | 0.0441 | 0.0395 | 0.0509 |  | 1.35 | 0.0004 | 1.21 | 0.0068 | 1.56 | 0.0015 |
| 291.25002 | 147 | Lactic acid | 4.9228 | 3.8157 | 3.6700 | 4.0344 |  | 0.78 | <0.0001 | 0.75 | 0.0002 | 0.82 | 0.0096 |
| 303.10002 | 205 | Glycolic acid | 0.0011 | 0.0012 | 0.0012 | 0.0013 |  | 1.09 | 0.134 | 1.06 | 0.2946 | 1.15 | 0.1469 |
| 321.70002 | 116 | Alanine(2TMS) | 1.7616 | 1.6749 | 1.7153 | 1.6142 |  | 0.95 | 0.6236 | 0.97 | 0.8057 | 0.92 | 0.5491 |
| 334.99998 | 204 | Glycine(2TMS) | Minor |  |  |  |  |  |  |  |  |  |  |
| 337.09998 | 205 | 2-hydroxy-butyrate | 0.0073 | 0.0133 | 0.0116 | 0.0159 |  | 1.83 | <0.0001 | 1.59 | <0.0001 | 2.18 | 0.0005 |
| 340.10004 | 100 | Oxalate | 0.0010 | 0.0019 | 0.0021 | 0.0017 |  | 1.85 | <0.0001 | 2.02 | <0.0001 | 1.60 | <0.0001 |
| 343.30002 | 202 | Ketovaline_2 | Minor |  |  |  |  |  |  |  |  |  |  |
| 345.79998 | 116 | Sarcosine | 0.0016 | 0.0026 | 0.0028 | 0.0021 |  | 1.56 | 0.0512 | 1.73 | 0.0239 | 1.30 | 0.4728 |
| 359.85000 | 117 | 3-hydroxy-butyrate | 0.1179 | 0.2319 | 0.1782 | 0.3123 |  | 1.97 | 0.0425 | 1.51 | 0.0422 | 2.65 | 0.2447 |
| 367.99998 | 131 | 2-aminobutyric acid | 0.0054 | 0.0059 | 0.0073 | 0.0037 |  | 1.08 | 0.4545 | 1.36 | 0.0073 | 0.68 | 0.0362 |
| 372.49998 | 200 | Ketoisoleucine_1 | 0.0022 | 0.0027 | 0.0026 | 0.0028 |  | 1.22 | 0.0079 | 1.17 | 0.0487 | 1.29 | 0.0151 |
| 385.45002 | 96 | Ketoisoleucine_2 | Minor |  |  |  |  |  |  |  |  |  |  |
| 395.95002 | 144 | Valine (2TMS) | 0.7912 | 0.8739 | 0.9397 | 0.7751 |  | 1.10 | 0.1286 | 1.19 | 0.0184 | 0.98 | 0.8314 |
| 418.24998 | 116 | Dihydroxyacetone | 0.0006 | 0.0006 | 0.0006 | 0.0007 |  | 1.14 | 0.0018 | 1.03 | 0.0307 | 1.30 | 0.0022 |
| 422.74998 | 116 | Serine(2TMS) | Minor |  |  |  |  |  |  |  |  |  |  |
| 428.05002 | 174 | 2-aminoethanol | 0.0355 | 0.0388 | 0.0399 | 0.0373 |  | 1.10 | 0.0731 | 1.12 | 0.0392 | 1.05 | 0.4976 |
| 428.25000 | 201 | n-caprylic acid | 0.0047 | 0.0051 | 0.0053 | 0.0047 |  | 1.08 | 0.3822 | 1.13 | 0.4159 | 1.00 | 0.5624 |
| 428.80002 | 147 | Glycerol | 0.5091 | 0.4956 | 0.4952 | 0.4962 |  | 0.97 | 0.9561 | 0.97 | 0.9186 | 0.97 | 0.9803 |
| 429.10002 | 299 | Phosphate | 0.4223 | 0.4970 | 0.4637 | 0.5469 |  | 1.18 | 0.0139 | 1.10 | 0.1235 | 1.30 | 0.0088 |
| 429.94998 | 158 | Leucine | 0.4279 | 0.4994 | 0.5205 | 0.4678 |  | 1.17 | 0.1135 | 1.22 | 0.0967 | 1.09 | 0.4197 |
| 443.25000 | 159 | Isoleucine | 0.0218 | 0.0294 | 0.0303 | 0.0280 |  | 1.35 | 0.0012 | 1.39 | 0.0043 | 1.29 | 0.0173 |
| 444.79998 | 130 | Threonine(2TMS) | Minor |  |  |  |  |  |  |  |  |  |  |
| 448.39998 | 142 | Proline | 0.4039 | 0.4629 | 0.4755 | 0.4439 |  | 1.15 | 0.1619 | 1.18 | 0.1581 | 1.10 | 0.4312 |
| 451.99998 | 174 | Glycine(3TMS) | 1.2896 | 1.4151 | 1.4473 | 1.3667 |  | 1.10 | 0.0585 | 1.12 | 0.0676 | 1.06 | 0.2407 |
| 456.15000 | 247 | Succinic acid (or aldehyde) | 0.0012 | 0.0013 | 0.0014 | 0.0012 |  | 1.05 | 0.7192 | 1.12 | 0.2876 | 0.94 | 0.479 |
| 462.15000 | 292 | Glyceric acid | 0.0021 | 0.0030 | 0.0031 | 0.0029 |  | 1.41 | <0.0001 | 1.44 | 0.0004 | 1.37 | 0.0026 |
| 476.80002 | 245 | Fumaric acid | 0.0009 | 0.0010 | 0.0010 | 0.0010 |  | 1.08 | 0.4419 | 1.07 | 0.5374 | 1.09 | 0.5231 |
| 479.74998 | 204 | Serine(3TMS) | 0.1352 | 0.1749 | 0.1908 | 0.1512 |  | 1.29 | 0.0651 | 1.41 | 0.0244 | 1.12 | 0.5963 |
| 483.19998 | 262 | Alanine(3TMS) | Minor |  |  |  |  |  |  |  |  |  |  |
| 484.40004 | 215 | Nonanoic acid(C9) | 0.0050 | 0.0035 | 0.0036 | 0.0033 |  | 0.70 | 0.003 | 0.72 | 0.0216 | 0.67 | 0.0099 |
| 494.00004 | 117 | Threonine(3TMS) | 0.0975 | 0.1099 | 0.1160 | 0.1007 |  | 1.13 | 0.5619 | 1.19 | 0.1061 | 1.03 | 0.3102 |
| 520.05000 | 248 | β-alanine | 0.0022 | 0.0029 | 0.0029 | 0.0028 |  | 1.28 | 0.0296 | 1.30 | 0.0798 | 1.25 | 0.0708 |
| 520.35000 | 104 | Hydrocinnamate | 0.0008 | 0.0011 | 0.0013 | 0.0007 |  | 1.29 | 0.3048 | 1.56 | 0.35 | 0.88 | 0.479 |
| 548.40000 | 133 | Malic acid | 0.0026 | 0.0031 | 0.0030 | 0.0033 |  | 1.21 | 0.0075 | 1.15 | 0.1292 | 1.30 | 0.0023 |
| 551.65002 | 217 | Threitol | 0.0016 | 0.0023 | 0.0025 | 0.0020 |  | 1.39 | 0.0512 | 1.50 | 0.0445 | 1.22 | 0.2962 |
| 555.94998 | 217 | meso-erythritol | 0.0182 | 0.0514 | 0.0546 | 0.0466 |  | 2.83 | 0.003 | 3.01 | 0.0065 | 2.57 | 0.0419 |
| 565.30002 | 267 | Acetylsalicylic acid | 0.0012 | 0.0006 | 0.0006 | 0.0007 |  | 0.52 | 0.3515 | 0.49 | 0.1095 | 0.58 | 0.7401 |
| 565.45002 | 232 | Aspartic acid | 0.0316 | 0.0503 | 0.0482 | 0.0534 |  | 1.59 | 0.0002 | 1.52 | 0.0032 | 1.69 | 0.0011 |
| 568.65000 | 176 | Methionine | 0.0342 | 0.0376 | 0.0399 | 0.0340 |  | 1.10 | 0.3355 | 1.17 | 0.0522 | 1.00 | 0.4728 |
| 569.30004 | 140 | trans-4-hydroxy-L-proline | 0.0143 | 0.0172 | 0.0162 | 0.0187 |  | 1.21 | 0.0722 | 1.13 | 0.2245 | 1.31 | 0.0723 |
| 571.54998 | 156 | Pyroglutamic acid | 0.4411 | 0.5833 | 0.6330 | 0.5089 |  | 1.32 | <0.0001 | 1.44 | <0.0001 | 1.15 | 0.0419 |
| 579.04998 | 239 | Pyrogallol | 0.0010 | 0.0011 | 0.0011 | 0.0012 |  | 1.16 | 0.4296 | 1.14 | 0.7592 | 1.19 | 0.2783 |
| 586.65000 | 329 | Creatinine | 0.0010 | 0.0008 | 0.0008 | 0.0008 |  | 0.80 | 0.0042 | 0.78 | 0.0047 | 0.82 | 0.0895 |
| 594.00000 | 275 | 2-isopropylmalic acid | Internal standard | |  |  |  |  |  |  |  |  |  |
| 611.50002 | 85 | β-glutamic acid | 0.0077 | 0.0088 | 0.0078 | 0.0103 |  | 1.15 | 0.1124 | 1.02 | 0.7766 | 1.35 | 0.0088 |
| 614.44998 | 246 | Glutamic acid | 0.1138 | 0.2074 | 0.1756 | 0.2550 |  | 1.82 | <0.0001 | 1.54 | <0.0001 | 2.24 | <0.0001 |
| 617.70000 | 103 | Anthranilic acid | 0.0008 | 0.0010 | 0.0009 | 0.0011 |  | 1.18 | 0.4901 | 1.11 | 0.8887 | 1.28 | 0.2653 |
| 622.69998 | 218 | Phenylalanine | 0.1588 | 0.2242 | 0.2125 | 0.2417 |  | 1.41 | <0.0001 | 1.34 | <0.0001 | 1.52 | 0.0003 |
| 623.40000 | 223 | p-hydroxybenzoic acid | 0.0014 | 0.0025 | 0.0027 | 0.0023 |  | 1.77 | <0.0001 | 1.89 | <0.0001 | 1.58 | 0.0004 |
| 624.40002 | 217 | Lyxose_1(or Xylose_1) | Minor |  |  |  |  |  |  |  |  |  |  |
| 627.40002 | 103 | Xylose_2 | 0.0046 | 0.0061 | 0.0074 | 0.0042 |  | 1.34 | 0.7231 | 1.62 | 0.4073 | 0.91 | 0.6811 |
| 628.29996 | 179 | 4-hydroxyphenylacetic acid | 0.0007 | 0.0008 | 0.0007 | 0.0011 |  | 1.27 | 0.1313 | 1.02 | 0.8116 | 1.64 | 0.0114 |
| 629.14998 | 117 | Lyxose_2 | 0.0010 | 0.0014 | 0.0013 | 0.0015 |  | 1.41 | 0.1424 | 1.31 | 0.5525 | 1.56 | 0.0471 |
| 630.69996 | 103 | Threo-β-hydroxyaspartic acid | 0.0047 | 0.0068 | 0.0064 | 0.0074 |  | 1.46 | 0.0002 | 1.38 | 0.0048 | 1.58 | 0.0007 |
| 630.94998 | 217 | Arabinose | 0.0018 | 0.0029 | 0.0028 | 0.0030 |  | 1.65 | <0.0001 | 1.61 | <0.0001 | 1.71 | <0.0001 |
| 633.64998 | 257 | Lauric acid | 0.0123 | 0.0152 | 0.0162 | 0.0138 |  | 1.24 | 0.0016 | 1.31 | 0.0005 | 1.12 | 0.1672 |
| 635.35002 | 98 | N-acetyl-L-aspartic acid_1 | Minor |  |  |  |  |  |  |  |  |  |  |
| 637.30002 | 89 | Ribulose | 0.0040 | 0.0030 | 0.0026 | 0.0036 |  | 0.75 | 0.0389 | 0.65 | 0.0064 | 0.89 | 0.7476 |
| 637.85004 | 217 | Ribose | 0.0159 | 0.0124 | 0.0112 | 0.0141 |  | 0.78 | 0.0837 | 0.70 | 0.0258 | 0.89 | 0.7327 |
| 638.80002 | 231 | Asparagine | 0.0138 | 0.0161 | 0.0168 | 0.0151 |  | 1.17 | 0.047 | 1.22 | 0.0422 | 1.10 | 0.2783 |
| 641.25000 | 326 | Taurine | 0.0048 | 0.0184 | 0.0263 | 0.0066 |  | 3.88 | 0.0024 | 5.54 | <0.0001 | 1.39 | 0.7327 |
| 650.25000 | 217 | Xylitol | 0.0022 | 0.0029 | 0.0027 | 0.0033 |  | 1.34 | 0.0009 | 1.22 | 0.0445 | 1.51 | 0.0003 |
| 652.75002 | 147 | Phthalic acid | 0.0030 | 0.0034 | 0.0030 | 0.0041 |  | 1.15 | 0.8935 | 0.99 | 0.1078 | 1.38 | 0.0648 |
| 655.89996 | 204 | 1,6-anhydroglucose | 0.0008 | 0.0006 | 0.0007 | 0.0005 |  | 0.80 | 0.3024 | 0.88 | 0.6042 | 0.68 | 0.2068 |
| 656.80002 | 103 | Arabitol | 0.0348 | 0.0408 | 0.0332 | 0.0522 |  | 1.17 | 0.0592 | 0.95 | 0.658 | 1.50 | 0.0029 |
| 659.14998 | 217 | Ribitol | 0.0018 | 0.0023 | 0.0021 | 0.0025 |  | 1.28 | 0.2975 | 1.19 | 0.3196 | 1.43 | 0.5103 |
| 661.40004 | 160 | Rhamnose_2 | Minor |  |  |  |  |  |  |  |  |  |  |
| 671.85000 | 103 | Putrescine | 0.0007 | 0.0007 | 0.0006 | 0.0008 |  | 0.89 | 0.0097 | 0.77 | 0.0014 | 1.08 | 0.4976 |
| 672.85002 | 229 | Aconitate | 0.0006 | 0.0007 | 0.0008 | 0.0007 |  | 1.15 | 0.043 | 1.18 | 0.0331 | 1.10 | 0.3055 |
| 685.89996 | 156 | Glutamine | 1.3986 | 1.2105 | 1.2599 | 1.1364 |  | 0.87 | 0.1112 | 0.90 | 0.5274 | 0.81 | 0.0298 |
| 687.19998 | 267 | 4-hydroxymandelate | 0.0006 | 0.0006 | 0.0006 | 0.0007 |  | 0.99 | 0.5584 | 0.93 | 0.2303 | 1.08 | 0.6452 |
| 687.40002 | 209 | Methoxy-4-hydroxyphenylacetate | 0.0007 | 0.0006 | 0.0006 | 0.0006 |  | 0.89 | 0.1286 | 0.89 | 0.2363 | 0.89 | 0.1985 |
| 689.05002 | 100 | O-phosphoethanolamine | 0.0021 | 0.0019 | 0.0019 | 0.0020 |  | 0.91 | 0.0161 | 0.89 | 0.0104 | 0.94 | 0.2367 |
| 701.85000 | 248 | Glycyl-glycine_1 | 0.0012 | 0.0012 | 0.0011 | 0.0012 |  | 1.00 | 0.368 | 0.98 | 0.3233 | 1.04 | 0.6884 |
| 702.30000 | 273 | Citric acid + isocitric acid | 0.1823 | 0.2258 | 0.2306 | 0.2185 |  | 1.24 | 0.0012 | 1.27 | 0.002 | 1.20 | 0.038 |
| 703.95000 | 200 | Ornithine | 0.0323 | 0.0406 | 0.0417 | 0.0390 |  | 1.26 | 0.0062 | 1.29 | 0.0042 | 1.21 | 0.1583 |
| 705.40002 | 265 | Hypoxanthine | 0.0124 | 0.0131 | 0.0132 | 0.0129 |  | 1.06 | 0.5101 | 1.07 | 0.4745 | 1.04 | 0.7627 |
| 707.29998 | 157 | Citrulline | 0.0038 | 0.0044 | 0.0044 | 0.0044 |  | 1.15 | 0.0458 | 1.16 | 0.1078 | 1.14 | 0.0972 |
| 711.90000 | 217 | Tagatose_1 | Minor |  |  |  |  |  |  |  |  |  |  |
| 715.95000 | 217 | Psicose_1 | Minor |  |  |  |  |  |  |  |  |  |  |
| 718.20000 | 147 | 1,5-anhydro-D-glucitol | 0.4647 | 0.3850 | 0.4450 | 0.2950 |  | 0.83 | 0.0674 | 0.96 | 0.8588 | 0.63 | 0.0013 |
| 720.40002 | 217 | Tagatose_2 (or Psicose_2) | 0.0028 | 0.0036 | 0.0035 | 0.0039 |  | 1.29 | 0.0915 | 1.23 | 0.2981 | 1.37 | 0.0692 |
| 722.29998 | 200 | Lysine(3TMS) | Minor |  |  |  |  |  |  |  |  |  |  |
| 722.55000 | 217 | α-sorbopyranose_1 (or Fructose_1) | 0.0207 | 0.0386 | 0.0279 | 0.0546 |  | 1.86 | <0.0001 | 1.35 | <0.0001 | 2.63 | <0.0001 |
| 723.75000 | 206 | Hippurate_2 | Minor |  |  |  |  |  |  |  |  |  |  |
| 726.55002 | 217 | Fructose_2 | Minor |  |  |  |  |  |  |  |  |  |  |
| 728.90004 | 147 | Mannose_1 | 0.1397 | 0.2194 | 0.1852 | 0.2707 |  | 1.57 | <0.0001 | 1.33 | <0.0001 | 1.94 | <0.0001 |
| 729.79998 | 176 | 5-dehydroquinic acid | 0.0005 | 0.0007 | 0.0006 | 0.0008 |  | 1.51 | <0.0001 | 1.35 | 0.0039 | 1.75 | <0.0001 |
| 733.80000 | 205 | Glucose_1 | 5.1614 | 4.2679 | 3.9857 | 4.6912 |  | 0.83 | 0.001 | 0.77 | 0.0008 | 0.91 | 0.0634 |
| 736.54998 | 147 | Allose_2 | Minor |  |  |  |  |  |  |  |  |  |  |
| 737.55000 | 133 | Sebacic acid | 0.0021 | 0.0019 | 0.0018 | 0.0021 |  | 0.94 | 0.0905 | 0.89 | 0.0785 | 1.01 | 0.3755 |
| 740.85000 | 205 | Galactose_2 | Minor |  |  |  |  |  |  |  |  |  |  |
| 742.65000 | 147 | Glucose_2 | Minor |  |  |  |  |  |  |  |  |  |  |
| 747.55002 | 217 | Gulcono-1,4-lactone | 0.0225 | 0.0600 | 0.0433 | 0.0851 |  | 2.67 | 0.0585 | 1.92 | 0.0378 | 3.78 | 0.3972 |
| 747.65004 | 156 | Lysine(4TMS) | 1.3406 | 1.2576 | 1.3248 | 1.1569 |  | 0.94 | 0.2408 | 0.99 | 0.8351 | 0.86 | 0.0128 |
| 749.20002 | 155 | Histidine | 0.0301 | 0.0392 | 0.0489 | 0.0247 |  | 1.30 | 0.8481 | 1.62 | 0.1217 | 0.82 | 0.0168 |
| 749.80002 | 205 | Galactosamine_1 | 0.0034 | 0.0048 | 0.0048 | 0.0047 |  | 1.40 | 0.0093 | 1.41 | 0.139 | 1.37 | 0.003 |
| 750.30000 | 217 | Glucuronate_1 | 0.0045 | 0.0053 | 0.0051 | 0.0056 |  | 1.17 | 0.0029 | 1.12 | 0.0422 | 1.23 | 0.0032 |
| 752.10000 | 129 | Glucosamine_2 | 0.0036 | 0.0040 | 0.0040 | 0.0040 |  | 1.13 | 0.0003 | 1.12 | 0.0078 | 1.13 | 0.0007 |
| 754.15002 | 205 | Galactosamine_2 | Minor |  |  |  |  |  |  |  |  |  |  |
| 756.30000 | 218 | Tyrosine | 0.4698 | 0.5854 | 0.6184 | 0.5358 |  | 1.25 | 0.0004 | 1.32 | 0.0002 | 1.14 | 0.0708 |
| 757.30002 | 332 | Ascorbic acid | 0.0007 | 0.0011 | 0.0011 | 0.0009 |  | 1.45 | 0.9226 | 1.55 | 0.8116 | 1.29 | 0.6171 |
| 757.75002 | 89 | Glucuronate_2 | Minor |  |  |  |  |  |  |  |  |  |  |
| 760.39998 | 281 | Gallic acid | 0.0007 | 0.0007 | 0.0006 | 0.0008 |  | 0.96 | 0.5619 | 0.87 | 0.2274 | 1.09 | 0.6311 |
| 765.60000 | 299 | 1-hexadecanol | 0.0019 | 0.0022 | 0.0022 | 0.0023 |  | 1.15 | 0.0082 | 1.14 | 0.0385 | 1.17 | 0.0231 |
| 766.70004 | 218 | Coniferyl aldehyde_2 | Minor |  |  |  |  |  |  |  |  |  |  |
| 767.34996 | 172 | N-α-acetyl-L-ornithine_1 | Minor |  |  |  |  |  |  |  |  |  |  |
| 775.20000 | 237 | Paraxanthine | 0.0039 | 0.0050 | 0.0060 | 0.0033 |  | 1.27 | 0.5514 | 1.55 | 0.1078 | 0.85 | 0.3294 |
| 779.59998 | 149 | N-α-acetyl-L-ornithine_2 | 0.0012 | 0.0015 | 0.0014 | 0.0017 |  | 1.20 | 0.069 | 1.11 | 0.6042 | 1.34 | 0.006 |
| 781.74996 | 218 | S-benzyl-L-cysteine_1 | 0.0007 | 0.0009 | 0.0008 | 0.0010 |  | 1.17 | 0.0202 | 1.02 | 0.0687 | 1.40 | 0.046 |
| 792.34998 | 95 | Palmitoleate | 0.0032 | 0.0041 | 0.0042 | 0.0039 |  | 1.27 | 0.043 | 1.31 | 0.0239 | 1.20 | 0.3917 |
| 810.94998 | 174 | Dopamine | 0.0006 | 0.0012 | 0.0010 | 0.0015 |  | 2.04 | <0.0001 | 1.66 | 0.0015 | 2.61 | <0.0001 |
| 812.59998 | 147 | Inositol | 0.1300 | 0.1538 | 0.1551 | 0.1518 |  | 1.18 | 0.0178 | 1.19 | 0.0093 | 1.17 | 0.2871 |
| 815.79996 | 315 | Arabinose-5-phosphate_2 | Minor |  |  |  |  |  |  |  |  |  |  |
| 816.25002 | 441 | Uric acid | 0.5360 | 0.5273 | 0.5448 | 0.5011 |  | 0.98 | 0.5409 | 1.02 | 0.9487 | 0.93 | 0.2871 |
| 823.54998 | 174 | N-α-acetyl-L-lysine_2 | 0.0102 | 0.0183 | 0.0107 | 0.0297 |  | 1.80 | 0.0115 | 1.05 | 0.4379 | 2.92 | 0.0002 |
| 836.70000 | 327 | Heptadecanoate | 0.0006 | 0.0008 | 0.0008 | 0.0009 |  | 1.40 | <0.0001 | 1.37 | <0.0001 | 1.44 | <0.0001 |
| 853.95000 | 218 | Kynurenine | 0.0007 | 0.0013 | 0.0012 | 0.0014 |  | 1.83 | <0.0001 | 1.74 | <0.0001 | 1.96 | <0.0001 |
| 859.80000 | 100 | Cysteamine+cystamine | 0.0301 | 0.0430 | 0.0473 | 0.0365 |  | 1.43 | <0.0001 | 1.57 | <0.0001 | 1.21 | 0.0755 |
| 864.00000 | 202 | Tryptophan | 0.1342 | 0.1202 | 0.1320 | 0.1025 |  | 0.90 | 0.0329 | 0.98 | 0.429 | 0.76 | 0.0028 |
| 865.75002 | 95 | Elaidic acid | 0.0020 | 0.0023 | 0.0023 | 0.0022 |  | 1.11 | 0.2761 | 1.12 | 0.2188 | 1.09 | 0.6595 |
| 887.34996 | 218 | Cysteine+cysteine | 0.0328 | 0.0507 | 0.0592 | 0.0380 |  | 1.55 | <0.0001 | 1.81 | <0.0001 | 1.16 | 0.229 |
| 937.35000 | 129 | 2'-deoxyuridine_2 | 0.0017 | 0.0025 | 0.0023 | 0.0029 |  | 1.51 | <0.0001 | 1.37 | 0.0014 | 1.72 | 0.0016 |
| 1025.50002 | 204 | Lactitol | 0.0003 | 0.0023 | 0.0004 | 0.0052 |  | 7.44 | 0.0316 | 1.16 | 0.5274 | 16.87 | 0.0013 |
| 1027.05000 | 204 | Maltose_2 | Minor |  |  |  |  |  |  |  |  |  |  |

The metabolites detected in human serum along with their retention times (RT) and quantitative ions (Q-ion) are listed in Table S4. In GC/MS analysis, multiple peaks are sometimes detected for a particular metabolite due to TMS-derivatization, isomeric form, etc. In such cases, each metabolite had the term ‘_1’, ‘_2’, or ‘(-TMS)’ added to the end of its name, according to the method described in a previous report [7]. In addition, the peak that most closely reflected the level of the metabolite was adopted for the subsequent evaluation, and the excluded peaks are indicated by the term ‘Minor’ in Table S4. In the training set, relative peak intensity values compared with that of the internal standard are shown for both the healthy volunteers and colorectal cancer patients with stage 0-4, stage 0-2, or stage 3-4 disease. In addition, the concentration of each metabolite in the colorectal cancer patients with stage 0-4, stage 0-2, or stage 3-4 disease was compared with that detected in the healthy volunteers, and the fold induction was calculated. P values were calculated using the Mann-Whitney U test, and p values of less than 0.05 were considered to indicate a significant difference.
