# Supplementary material for: A Novel Serum Metabolomics-Based Diagnostic Approach for Colorectal Cancer
Source: PLoS One. 2012 Jul 11;7(7):e40459. doi: 10.1371/journal.pone.0040459 (PMC3394708; doi:10.1371/journal.pone.0040459)
Supplement: Table S5 — Comparison of serum metabolite levels between the colorectal cancer patients and healthy volunteers in the data set mixing the training set with the validation set. (DOC) [file pone.0040459.s008.doc]

**Table S5.** Comparison of serum metabolite levels between the colorectal cancer patients and healthy volunteers in the data set mixing the training set with the validation set

|  | Colorectal cancer patients | | | | | | | |
| --- | --- | --- | --- | --- | --- | --- | --- | --- |
|  | Stage 0-4 | |  | Stage 0-2 | |  | Stage 3-4 | |
|  | Fold induction | p value |  | Fold induction | p value |  | Fold induction | p value |
| Pyruvate+Oxalacetic acid | 1.44 | <0.0001 |  | 1.36 | <0.0001 |  | 1.53 | <0.0001 |
| Lactic acid | 0.78 | <0.0001 |  | 0.74 | <0.0001 |  | 0.83 | <0.0001 |
| Glycolic acid | 1.13 | 0.0083 |  | 1.06 | 0.0791 |  | 1.22 | 0.0095 |
| Alanine(2TMS) | 1.02 | 0.4603 |  | 0.98 | 0.9259 |  | 1.06 | 0.1653 |
| 2-hydroxy-butyrate | 1.42 | <0.0001 |  | 1.35 | <0.0001 |  | 1.51 | 0.0003 |
| Oxalate | 1.70 | <0.0001 |  | 1.91 | <0.0001 |  | 1.45 | <0.0001 |
| Sarcosine | 1.54 | 0.0008 |  | 1.41 | 0.0427 |  | 1.69 | 0.0004 |
| 3-hydroxy-butyrate | 1.88 | 0.0097 |  | 1.54 | 0.0170 |  | 2.30 | 0.0741 |
| 2-aminobutyric acid | 1.03 | 0.6956 |  | 1.18 | 0.0821 |  | 0.86 | 0.1986 |
| Ketoisoleucine_1 | 1.17 | 0.0009 |  | 1.11 | 0.0740 |  | 1.25 | 0.0002 |
| Valine(2TMS) | 1.11 | 0.0078 |  | 1.09 | 0.0445 |  | 1.14 | 0.0193 |
| Dihydroxyacetone | 1.23 | <0.0001 |  | 1.09 | 0.0151 |  | 1.39 | <0.0001 |
| 2-aminoethanol | 1.13 | 0.0018 |  | 1.12 | 0.0059 |  | 1.15 | 0.0210 |
| n-caprylic acid | 1.13 | 0.0124 |  | 1.17 | 0.0147 |  | 1.07 | 0.1144 |
| Glycerol | 1.06 | 0.2450 |  | 1.00 | 0.6120 |  | 1.13 | 0.1494 |
| Phosphate | 1.16 | 0.0021 |  | 1.06 | 0.2321 |  | 1.27 | <0.0001 |
| Leucine | 1.16 | 0.0035 |  | 1.11 | 0.1178 |  | 1.23 | 0.0010 |
| Isoleucine | 1.35 | <0.0001 |  | 1.26 | 0.0081 |  | 1.46 | <0.0001 |
| Proline | 1.23 | 0.0016 |  | 1.16 | 0.0621 |  | 1.33 | 0.0007 |
| Glycine(3TMS) | 1.16 | 0.0001 |  | 1.16 | 0.0018 |  | 1.15 | 0.0190 |
| Succinic acid(or aldehyde) | 1.11 | 0.0676 |  | 1.12 | 0.0791 |  | 1.09 | 0.2366 |
| Glyceric acid | 1.30 | <0.0001 |  | 1.31 | <0.0001 |  | 1.29 | <0.0001 |
| Fumaric acid | 1.08 | 0.2050 |  | 1.05 | 0.5465 |  | 1.12 | 0.1298 |
| Serine(3TMS) | 1.36 | <0.0001 |  | 1.32 | 0.0089 |  | 1.42 | 0.0001 |
| Nonanoic acid(C9) | 0.78 | 0.0057 |  | 0.79 | 0.0300 |  | 0.77 | 0.0193 |
| Threonine(3TMS) | 1.09 | 0.1966 |  | 1.05 | 0.2445 |  | 1.14 | 0.3564 |
| β-Alanine | 1.30 | 0.0004 |  | 1.33 | 0.0031 |  | 1.26 | 0.0057 |
| Hydrocinnamate | 1.28 | 0.2641 |  | 1.33 | 0.5427 |  | 1.23 | 0.2123 |
| Malic acid | 1.37 | <0.0001 |  | 1.28 | 0.0007 |  | 1.47 | <0.0001 |
| Threitol | 1.35 | 0.0006 |  | 1.38 | 0.0050 |  | 1.32 | 0.0061 |
| meso-erythritol | 2.01 | <0.0001 |  | 2.03 | <0.0001 |  | 1.99 | 0.0001 |
| Acetylsalicylic acid | 0.81 | 0.1330 |  | 0.64 | 0.0439 |  | 1.00 | 0.7440 |
| Aspartic acid | 1.68 | <0.0001 |  | 1.47 | <0.0001 |  | 1.93 | <0.0001 |
| Methionine | 1.08 | 0.1442 |  | 1.04 | 0.2537 |  | 1.12 | 0.2146 |
| trans-4-hydroxy-L-proline | 1.15 | 0.0050 |  | 1.02 | 0.2160 |  | 1.32 | 0.0005 |
| Pyroglutamic acid | 1.35 | <0.0001 |  | 1.44 | <0.0001 |  | 1.25 | <0.0001 |
| Pyrogallol | 1.10 | 0.9883 |  | 0.97 | 0.1459 |  | 1.26 | 0.0947 |
| Creatinine | 0.90 | 0.0190 |  | 0.85 | 0.0071 |  | 0.96 | 0.3057 |
| β-Glutamic acid | 1.13 | 0.0290 |  | 1.08 | 0.3539 |  | 1.19 | 0.0062 |
| Glutamic acid | 1.94 | <0.0001 |  | 1.57 | <0.0001 |  | 2.38 | <0.0001 |
| Anthranilic acid | 1.14 | 0.7801 |  | 1.07 | 0.8194 |  | 1.22 | 0.4569 |
| Phenylalanine | 1.41 | <0.0001 |  | 1.26 | <0.0001 |  | 1.59 | <0.0001 |
| p-hydroxybenzoic acid | 1.60 | <0.0001 |  | 1.65 | <0.0001 |  | 1.53 | <0.0001 |
| Xylose_2 | 1.33 | 0.0230 |  | 1.49 | 0.0941 |  | 1.15 | 0.0404 |
| 4-hydroxyphenylacetic acid | 1.22 | 0.3890 |  | 1.08 | 0.7846 |  | 1.39 | 0.2366 |
| Lyxose_2 | 1.43 | 0.0943 |  | 1.36 | 0.6319 |  | 1.52 | 0.0006 |
| Threo-β-hydroxyaspartic acid | 1.50 | <0.0001 |  | 1.45 | <0.0001 |  | 1.55 | <0.0001 |
| Arabinose | 1.52 | <0.0001 |  | 1.48 | <0.0001 |  | 1.56 | <0.0001 |
| Lauric acid | 1.25 | <0.0001 |  | 1.33 | <0.0001 |  | 1.15 | 0.0007 |
| Ribulose | 0.71 | 0.0006 |  | 0.66 | 0.0003 |  | 0.78 | 0.0696 |
| Ribose | 0.71 | 0.0002 |  | 0.66 | 0.0002 |  | 0.76 | 0.0285 |
| Asparagine | 1.17 | 0.0069 |  | 1.13 | 0.0851 |  | 1.23 | 0.0061 |
| Taurine | 3.43 | <0.0001 |  | 4.63 | <0.0001 |  | 1.99 | 0.1080 |
| Xylitol | 1.36 | <0.0001 |  | 1.28 | 0.0108 |  | 1.46 | <0.0001 |
| Phthalic acid | 1.19 | 0.9531 |  | 1.14 | 0.2669 |  | 1.25 | 0.1742 |
| 1,6-anhydroglucose | 0.97 | 0.2289 |  | 0.97 | 0.9326 |  | 0.96 | 0.0290 |
| Arabitol | 1.35 | 0.0002 |  | 1.31 | 0.0416 |  | 1.39 | <0.0001 |
| Ribitol | 1.42 | 0.0042 |  | 1.35 | 0.0134 |  | 1.49 | 0.0302 |
| Putrescine | 0.87 | 0.0001 |  | 0.72 | <0.0001 |  | 1.05 | 0.1331 |
| Aconitate | 1.20 | 0.0004 |  | 1.20 | 0.0160 |  | 1.21 | 0.0095 |
| Glutamine | 0.90 | 0.0721 |  | 0.90 | 0.1699 |  | 0.91 | 0.1174 |
| 4-hydroxymandelate | 1.01 | 0.0618 |  | 0.91 | 0.0363 |  | 1.13 | 0.3836 |
| Methoxy-4-hydroxyphenylacetate | 0.89 | 0.0008 |  | 0.80 | 0.0015 |  | 0.99 | 0.0244 |
| O-phosphoethanolamine | 0.95 | 0.0041 |  | 0.92 | 0.0009 |  | 0.98 | 0.2158 |
| Glycyl-Glycine_1 | 1.14 | 0.1113 |  | 1.08 | 0.6219 |  | 1.22 | 0.0276 |
| Citric acid + Isocitric acid | 1.28 | <0.0001 |  | 1.28 | <0.0001 |  | 1.28 | 0.0004 |
| Ornithine | 1.37 | <0.0001 |  | 1.36 | <0.0001 |  | 1.37 | <0.0001 |
| Hypoxanthine | 0.99 | 0.6395 |  | 1.04 | 0.7416 |  | 0.92 | 0.2353 |
| Citrulline | 1.18 | 0.0001 |  | 1.09 | 0.1086 |  | 1.29 | <0.0001 |
| 1,5-anhydro-D-glucitol | 0.87 | 0.0483 |  | 1.04 | 0.3717 |  | 0.67 | <0.0001 |
| Tagatose_2(or Psicose_2) | 1.68 | 0.0007 |  | 1.57 | 0.0159 |  | 1.82 | 0.0016 |
| α-sorbopyranose_1(or Fructose_1) | 1.57 | <0.0001 |  | 1.13 | <0.0001 |  | 2.10 | <0.0001 |
| Mannose_1 | 1.52 | <0.0001 |  | 1.26 | <0.0001 |  | 1.83 | <0.0001 |
| 5-dehydroquinic acid | 1.47 | <0.0001 |  | 1.23 | 0.0034 |  | 1.77 | <0.0001 |
| Glucose_1 | 0.82 | <0.0001 |  | 0.73 | <0.0001 |  | 0.93 | 0.0006 |
| Sebacic acid | 1.02 | 0.1557 |  | 0.96 | 0.0947 |  | 1.10 | 0.5674 |
| Gulcono-1,4-lactone | 2.04 | 0.0051 |  | 1.88 | 0.0071 |  | 2.23 | 0.0701 |
| Lysine(4TMS) | 0.94 | 0.0907 |  | 0.98 | 0.9708 |  | 0.89 | 0.0036 |
| Histidine | 1.14 | 0.3971 |  | 1.31 | 0.0745 |  | 0.93 | 0.5848 |
| Galactosamine_1 | 1.57 | <0.0001 |  | 1.59 | 0.0012 |  | 1.54 | <0.0001 |
| Glucuronate_1 | 1.34 | <0.0001 |  | 1.34 | <0.0001 |  | 1.34 | <0.0001 |
| Glucosamine_2 | 1.28 | <0.0001 |  | 1.28 | <0.0001 |  | 1.28 | <0.0001 |
| Tyrosine | 1.25 | <0.0001 |  | 1.27 | <0.0001 |  | 1.22 | 0.0002 |
| Ascorbic acid | 1.15 | 0.5854 |  | 1.30 | 0.9730 |  | 0.97 | 0.3647 |
| Gallic acid | 0.90 | 0.0020 |  | 0.81 | 0.0005 |  | 1.01 | 0.1441 |
| 1-hexadecanol | 1.18 | <0.0001 |  | 1.15 | 0.0057 |  | 1.21 | 0.0001 |
| ParaXanthine | 1.24 | 0.7423 |  | 1.33 | 0.6120 |  | 1.14 | 0.9987 |
| N-α-acetyl-L-Ornithine_2 | 1.30 | 0.0018 |  | 1.20 | 0.0695 |  | 1.41 | 0.0008 |
| S-benzyl-L-Cysteine_1 | 1.19 | 0.0001 |  | 1.02 | 0.0026 |  | 1.41 | 0.0009 |
| Palmitoleate | 1.35 | 0.0049 |  | 1.44 | 0.0053 |  | 1.24 | 0.0836 |
| Dopamine | 1.97 | <0.0001 |  | 1.96 | <0.0001 |  | 1.99 | <0.0001 |
| Inositol | 1.19 | <0.0001 |  | 1.20 | <0.0001 |  | 1.17 | 0.0106 |
| Uric acid | 1.02 | 0.8456 |  | 1.03 | 0.7437 |  | 1.00 | 0.9759 |
| N-α-acetyl-L-Lysine_2 | 1.68 | 0.0041 |  | 1.34 | 0.4042 |  | 2.08 | <0.0001 |
| Heptadecanoate | 1.33 | <0.0001 |  | 1.30 | 0.0001 |  | 1.37 | 0.0002 |
| Kynurenine | 1.83 | <0.0001 |  | 1.72 | <0.0001 |  | 1.98 | <0.0001 |
| Cystamine | 1.34 | <0.0001 |  | 1.43 | <0.0001 |  | 1.24 | 0.0002 |
| Tryptophan | 1.00 | 0.1292 |  | 1.03 | 0.7238 |  | 0.97 | 0.0256 |
| Elaidic acid | 1.14 | 0.0126 |  | 1.13 | 0.0322 |  | 1.15 | 0.0570 |
| Cysteine+Cystine | 1.72 | <0.0001 |  | 1.83 | <0.0001 |  | 1.58 | <0.0001 |
| 2'-deoxyuridine_2 | 1.52 | <0.0001 |  | 1.37 | 0.0006 |  | 1.71 | <0.0001 |
| Lactitol | 4.35 | 0.0026 |  | 1.17 | 0.5060 |  | 8.19 | <0.0001 |

In the data set mixing the training set with the validation set, the concentration of each metabolite in the colorectal cancer patients with stage 0-4, stage 0-2, or stage 3-4 disease was compared with that detected in the healthy volunteers, and the fold induction was calculated. P values were calculated using the Mann-Whitney U test, and p values of less than 0.05 were considered to indicate a significant difference.
